# Open-label placebo treatment does not enhance cognitive abilities in healthy volunteers

**DOI:** 10.1038/s41598-023-45979-3

**Published:** 2023-11-09

**Authors:** Helena Hartmann, Katarina Forkmann, Katharina Schmidt, Julian Kleine-Borgmann, Johanna Albers, Katja Wiech, Ulrike Bingel

**Affiliations:** 1 Clinical Neurosciences, Department of Neurology and Center for Translational Neuro- and Behavioral Sciences (C-TNBS), University Hospital Essen, Essen, Germany; 2 Nuffield Department of Clinical Neurosciences, John Radcliffe Hospital, University of Oxford, Haedington, Oxford, UK

**Keywords:** Human behaviour, Placebo effect

## Abstract

The use of so-called ‘smart drugs’ such as modafinil to improve cognitive performance has recently attracted considerable attention. However, their side effects have limited user enthusiasm. Open-label placebo (OLP) treatment, i.e., inert treatments that are openly disclosed to individuals as having no active pharmacological ingredient, has been shown to improve various medical symptoms and conditions, including those related to cognitive performance. OLP treatment could therefore be an exciting alternative to pharmacological cognitive enhancers. Here, we used a randomized-controlled design to investigate the effect of a 21-day OLP treatment on several sub-domains of cognitive performance in *N* = 78 healthy volunteers. Subjective and objective measures of cognitive performance as well as different measures of well-being were obtained before and after the treatment period. Using a combination of classic Frequentist and Bayesian analysis approaches showed no additional benefit from OLP treatment in any of the subjective or objective measures of cognitive performance. Our study thus highlights possible limitations of OLP treatment in boosting cognitive performance in healthy volunteers. These findings are discussed in the light of expectancy-value considerations that may determine OLP efficacy.

## Introduction

Cognitive performance is a vital aspect of daily functioning, and crucial for educational and occupational success^1,2^. Many clinical conditions are accompanied by cognitive impairments and daily stressors can compromise cognitive functioning in healthy samples^3,4^, which is often experienced as extremely debilitating. Those findings have conjured interest in enhancing individuals’ cognitive abilities to either alleviate existing symptoms or protect from developing such impairments. In recent years, ‘smart drugs’ such as modafinil have been employed to enhance cognitive performance, although these come with considerable side-effects^5,6^. While factors such as motivation, behavioural training and self-efficacy may be able to improve cognitive performance^7,8^, it has been suggested that positive treatment expectations and placebo effects in the absence of any pharmacological treatment may also be harnessed to improve different aspects of cognitive performance^9–11^.

For example, students with a positive treatment expectation showed improvements in a standard IQ test^9^ and Schwarz and Büchel^12^ found effects of allegedly different tone frequencies on subjective but not objective performance in a Flanker task. Similarly, Winkler and Hermann^10^ found that a placebo nasal spray that was expected to improve cognitive performance increased participants’ perceived but not their actual performance. Parong et al.^13^ showed that individuals with positive expectations improved more in a working memory task than those with negative expectations. Finally, expectations that heat pain would improve cognitive performance led to improved performance in a short-term memory task^14^. The findings of these studies illustrate the potential of positive expectations and non-pharmacological treatments to enhance cognitive performance in healthy samples but also give some indication of their limitations (e.g., that subjective gains may not be reflected in objective performance parameters). Moreover, these studies used so-called ‘deceptive’ placebos or expectancy manipulations in which participants are unaware of the (non-pharmacological) manipulation.

In search of strategies to overcome the ethical and legal limitations of deceptive placebos and increase trust in healthcare providers^15^, recent studies have explored the potential of ‘honest’ or open-label placebo (OLP) interventions, where individuals are fully informed about the inert nature of the given treatment^16^. OLP studies in patients (and some in healthy volunteers) have demonstrated improvements for a variety of outcomes including pain, allergies, fatigue, depression, attention deficits and hyperactivity, post-operative opioid consumption, and post-traumatic memory intrusions^17–19^. However, other studies in both clinical and healthy samples report no improvements through OLPs^20,21^ or only find effects in exploratory analyses^22,23^. These findings paint a more heterogeneous picture on the efficacy of OLPs, and highlight, that OLPs may enhance many aspects of functioning, but may not always work in all populations or against all symptoms.

So far, studies in healthy individuals assessing cognitive enhancement through OLPs are still scarce, but results of existing deceptive placebo studies point towards an interesting divide in this effect: While subjective markers (e.g., the subjective perception of one’s own performance) indicate improvements under OLP, this improvement is not reflected in objective performance parameters^10,12^. We recently investigated whether a 3-week OLP intake improves exam results of (stressed) medical students^24^. While OLPs had a positive effect on psychological well-being, we found no significant effect on actual exam performance. In line with this, a recent meta-analysis reported investigated OLP effects on mental and physical health in healthy volunteers and found a significant effect for subjective but not for objective outcomes^18^. Importantly, the level of suggestiveness of the instructions influenced the efficacy of OLPs for some outcomes. However, as previous studies only focused on a few selected outcome variables, a more comprehensive assessment of OLP effects on the broad spectrum of cognitive functions is still missing. This is crucial, as OLPs might only affect specific cognitive parameters in the sense of a “cognitive enhancement”, a hypothesis which previous studies were not able to assess fully. In this study we compared the effects of a three-week OLP treatment and no treatment in a group of healthy young adults on a large battery of subjective and objective measures of cognitive performance and well-being, focusing on validated, established measures with a specific focus on attention- and working memory-related facets of cognitive performance. Based on previous results from deceptive placebo studies, we hypothesized that the OLP treatment would lead to improvements in both subjective and objective indicators of cognitive performance and well-being, compared to a no-treatment control group.

## Materials and methods

### Clinical trial registration

The monocentric, randomized controlled study was registered in the German Clinical Trials Register on 30/10/2019 (Deutsches Register Klinischer Studien; DRKS; ID DRKS00019203; https://drks.de/search/de/trial/DRKS00019203).

### Ethics approval statement

All study procedures were approved by the University Duisburg-Essen Ethics Committee (19-8874-BO). The study was conducted in accordance with the principles of Good Clinical Practice^25^ and the Declaration of Helsinki^26^.

### Participants

Recruitment and data collection took place from October 2019 to March 2020 at the University Hospital Essen in Germany. Participants were recruited via flyers and posters at regional universities and the University Hospital Essen, via an in-house participant database and via social media. Those between 18 and 60 years of age were eligible to take part. Exclusion criteria comprised severe chronic or acute clinical conditions, regular substance use of cannabinoids, cocaine, or amphetamines in the last 4 weeks, color blindness, known allergies or intolerance against the ingredients of the placebo medication, pregnancy or breastfeeding, alcohol consumption on the testing days or the day before, or participation in other (medication-related) studies in the three weeks of testing. All participants underwent an initial phone interview and gave written, informed consent prior to participation. All participants received 50 € after study completion, plus the bonus gained in the Instrumental Learning Task (means and standard deviations are always reported as *M* ± *SD* = 62.03 ± 19.28 €; see text below for details on the task).

We collected data from 100 participants. Data of those participants who had only completed the Baseline assessment were discarded (*n* = 12; 4 from the OLP group). Additional datasets had to be excluded due to technical problems during the experimental sessions (*n* = 5; e.g. due to hardware or software malfunctioning) or because participants had taken the placebo medication less than 70% of the time (cut-off as recommended for the analysis of clinical trials^27^; *n* = 5). The final sample therefore comprised 78 participants, 40 participants in the OLP group (18 males, 22 females; *M*_age_ ± *SD* = 28.10 ± 10.46) and 38 participants in the control (CTR) group (15 males, 23 females; *M*_age_ ± *SD* = 28.82 ± 6.93). The two groups were comparable regarding age, gender, and educational background (all *p*’s > 0.189). We had conducted an *a-priori* power analysis based on results of our previous study (*N* = 154^24^). Using the package WebPower^28^ in RStudio (RStudio version 2022.07.0, build 548; R version 4.2.1^29^), we calculated that a sample size of *n* = 99 per group would be necessary to reach a Cohen’s *d* of 0.4 (2 groups, 2 measurements, *α* = 0.05, *β* = 0.8). Unfortunately, recruitment was stopped in March 2020 to comply with restrictions imposed by the German Government to prevent the spread of COVID-19. We decided not to restart the testing after the pandemic to avoid time- and experimenter-related effects^30^. An updated post hoc power calculation (for critiques of this approach see^31,32^) showed that with the current sample size of *N* = 78 participants who had been included up to this point, we had reached a power of 1-*β* = 0.41 (2 groups, 2 assessment time-points, Cohen’s *d* = 0.4 and *α* = 0.05). Due to this decreased power and thus a possibility of missing positive effects smaller than *d* = 0.4 with our sample size, we decided to add a Bayesian analysis framework to the results that can directly assess evidence for the null compared to the alternative hypothesis (see “Data acquisition and analysis” section).

### Procedure

**Figure 1:**
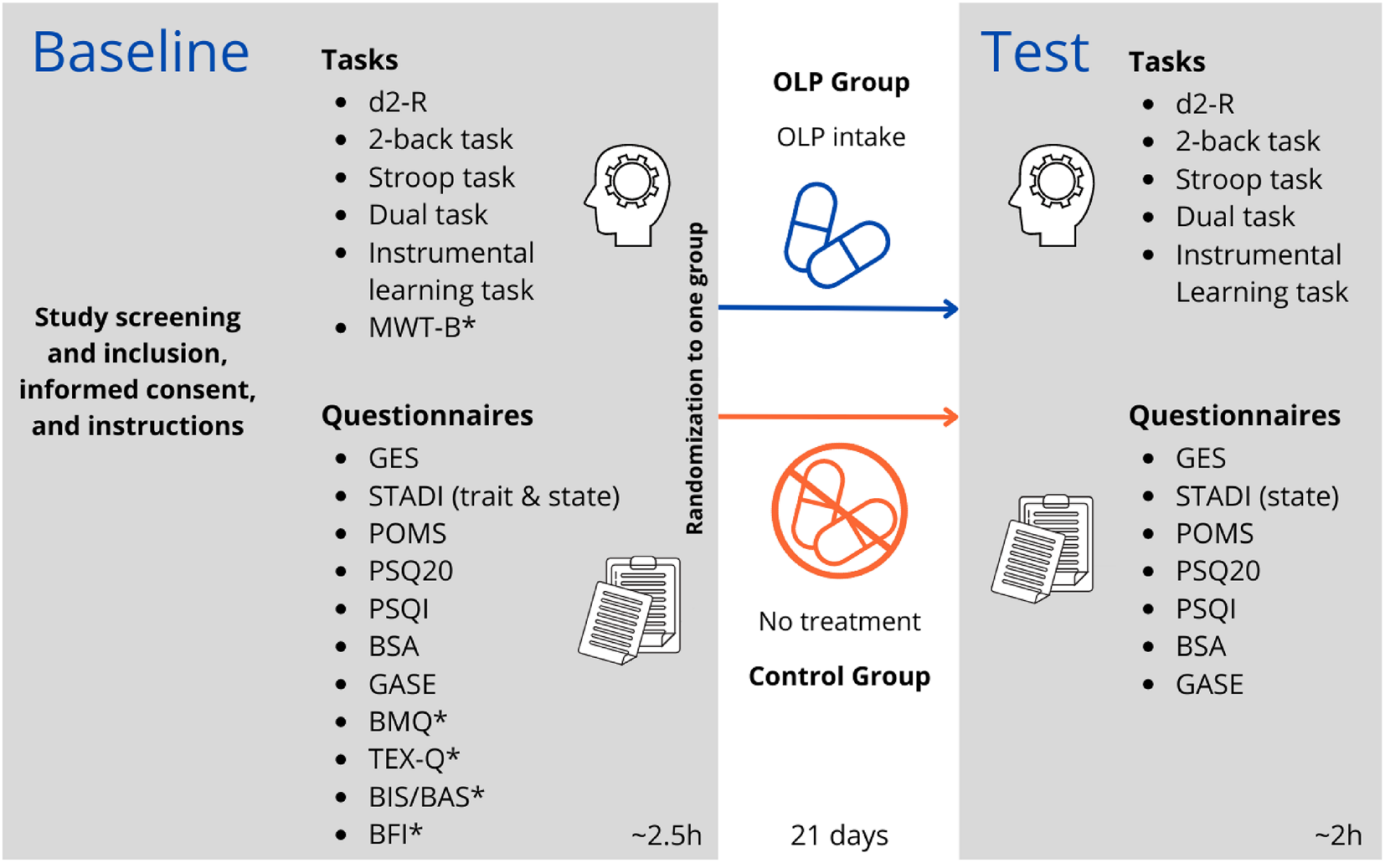
Overview of the study design. First, all tasks were completed before participants completed the questionnaires. Tests and questionnaires were then completed in German and in a randomized order, except the d2 which was administered first and the learning task which was always administered last. Trait questionnaires/tasks were only administered at the Baseline (*), all state measures were acquired twice. *d2-R* attention and concentration test, *MWT-B* multiple choice vocabulary intelligence test, *GES* health questionnaire, *STADI* state-trait-anxiety-depression-inventory, *POMS* profile of mood states, *PSQ20* perceived stress questionnaire, *PSQI* Pittsburg sleep quality index, *BSA* movement and sports activity questionnaire, *GASE* generic assessment of side effects, *BMQ* beliefs about medicines questionnaire, *TEX-Q* treatment expectation questionnaire, *BIS/BAS* behavioral inhibition/behavioral approach system questionnaire, *BFI* Big Five Inventory, *OLP* open-label placebo.

The study followed a single-blind, between-subjects design with two sessions (Baseline and Test; see also Fig. 1). Participants were randomly assigned to one of two groups with a pre-specified, computer-generated randomization list, while study experimenters, data analysts and data evaluators were not informed about group allocation until the end of the study (for flyers and participants’ instructions regarding the OLP treatment see Table S1 in the Supplement). At the Baseline session, all participants completed computer- and paper-based tests and questionnaires to assess cognitive performance and well-being (duration ca. 2.5 h; see below for details on all measures). Tests and questionnaires were completed in German and in a randomized order between-participants, except the d2 (paper–pencil), which was administered first and the learning task, which was always administered last (due to the use of a different PC operating system). At the end of the first session, each participant received a numbered 15 × 10 × 5 cm box from the study experimenter containing instructions for either the OLP or CTR group. Both box versions had been prepared by a third experimenter who was otherwise not involved in the study and were matched in weight and sound produced by handling the box.

Each box either contained 45 placebo pills (Zeebo Relief from Zeebo Effect, LCC; white-blue pills filled with silicon dioxide, titanium dioxide and microcrystalline cellulose) in their original packaging (OLP group), or filler material of similar weight but no pills (CTR group). Both groups also received a note that stated the group they had been allocated to, OLP intake instructions (OLP group) or the instructions to do nothing (CTR group) and a question asking about their satisfaction with the group assignment. We chose this approach to control for test–retest training effects over time. The OLP group was fully informed that the pills were placebos and instructed to take one tablet twice daily for 21 days, starting with the day after the Baseline session. This procedure and duration of the OLP intervention had been successfully established in previous work^24,33^, including the “pioneering” OLP studies^34,35^, and was therefore used to keep the resulting effects consistent and comparable (but see e.g.^10^ for a single administration of the OLP nasal spray). Between 22 and 27 days after the Baseline session, participants returned to the lab for the Test session (duration ca. 2 h). In both sessions, two participants were tested at the same time. Participants received noise-cancelling headphones for an undisturbed testing and sat opposite of each other with a partition wall between them. Participants were tested around the same time on each of their sessions (morning or afternoon) and on the same computer to minimize the influence of circadian fluctuations in cognitive performance^36^ and reduce hardware-related measurement differences of e.g. reaction time due to the computers’ processing speed.

### Baseline measures

At the Baseline session, we collected demographic data (age, gender, employment, years of education, medication intake and chronic conditions) from each participant as well as an in-house health questionnaire, including data on participants’ use of alcohol, caffeine, nicotine and other substances, to monitor changes in health status and validate the absence of exclusion criteria. To make sure the two groups were comparable in terms of intelligence, we administered the *Multiple Choice Vocabulary Intelligence Test* (MWT-B; paper–pencil test^37^). In each of the 37 items, participants are presented with five strings of letters and have to identify the one that constitutes a word. The sum of correctly identified words was recorded. The *Beliefs About Medicine Questionnaire* (BMQ^38^) asks participants to which extent they agree with 18 general statements about medication rated on a 5-point Likert scale from ‘agree completely’ to ‘disagree completely’. The *Treatment Expectation Questionnaire* (TEX-Q^39^) measures expectations related to treatments in 15 items that are answered on an 11-point Likert scales from ‘0—none’ to ‘10—highest imaginable’ on different dimensions (e.g., benefit, improvement, risk, burden, adverse effects, etc.). Participants were asked to imagine taking placebo pills for the next three weeks and answer the questionnaire with their expectations regarding this treatment in mind. The *Big Five Inventory* (BFI^40^) measures five personality characteristics with 10 items on a 5-point Likert scale from ‘does not apply at all’ to ‘applies completely’. The *Behavioral Inhibition and Behavioral Approach System Questionnaires* (BIS/BAS^41^) measures the inhibition vs. approach tendencies with 24 items rated on a 4-point Likert scale from ‘does not at all apply to me’ to ‘applies exactly to me’. The *Movement- and Sports Activity Questionnaire* (BSA^42^) uses 6 items to assess work and sport activity, where participants had to indicate the amount of days and minutes engaging in activities like bike riding and walking.

### Outcome measures

To investigate the effects of OLPs on cognitive performance, we aimed to assess a wide range of complex cognitive abilities while balancing feasibility, time constraints, and participant fatigue. We thus included five standardized, well-established tasks measuring the following aspects of cognitive performance, which have shown robust effects in previous work: mental processing conflicts (Stroop task), divided attention (Dual task), working memory (2-back task), learning of gains and losses (Instrumental learning task), and attention and concentration (d2 task).

Both groups received the same standardized instructions for each measure. All tasks and questionnaires were completed in German either on a computer or in a paper–pencil format, as indicated below. For all tasks, we excluded trials with reaction times below 200 ms^43^. We also excluded trials with reaction times smaller or larger than three standard deviations (SDs) from the participant’s individual mean^44^. For completeness and transparency, we report all outcomes measured in the study below, distinguishing preregistered primary and secondary outcomes from exploratory outcomes. The results of all exploratory outcomes are detailed in the Supplement. As preregistered, we conducted four tasks measuring different domains of cognitive performance. The d2 task was not preregistered but added later.

#### Objective measures

The *Stroop task* (computerized^45^) assesses mental processing conflicts and the level of interference between colors and letters. Colored words (“RED”, “BLUE”, “GREEN”, “YELLOW”) are presented on a screen for two seconds. In congruent trials these words are written in the same color as the word (e.g., a green-colored word “GREEN”), while they are written in a different color in incongruent trials (e.g., a blue-colored word “RED”). Participants had to indicate the color of the word as quickly as possible by pressing one of four keyboard keys. Allocation of colors to the four keys was pseudorandomized. A fixation cross was shown for two seconds between two consecutive words. In an initial training session, participants practiced the pressing of the buttons in response to colored squares and moved on to a test (20 trials) with feedback after 80% of the squares have been correctly identified. After this training, the actual task commenced with 96 trials presented in a randomized order (48 congruent, 48 incongruent, with each color and word shown the same number of times). An index for the Stroop effect was calculated as the difference in average reaction time between incongruent and congruent trials and recorded as primary outcome. Additional exploratory outcomes included the average reaction time for correct trials and accuracy (% correct responses), both calculated separately for congruent and incongruent trials.

The *dual task* (computerized^46^) measures how well people can divide their attention regarding visual and auditory stimuli. In two training sessions, participants attended to two tasks: (1) Seeing a grid of dots and x’s for 2 s and learning to press the left mouse button as fast as possible if four x’s form a square. (2) Hearing two tones (high or low pitch) and learning to press the left mouse button as fast as possible if the same tone is repeated twice (high-high or low-low). The first 10 trials included feedback on the accuracy, whereas the second 10 trials were presented without feedback. This was followed by the 100 trials of the main task (40 targets, 20 in the first half, 20 in the second half, with each 10 visual and 10 auditory targets). Visual and auditory targets were never presented simultaneously. As our primary outcome, we measured the average reaction time as well as the number of correct responses (hits), separately for visual and auditory targets. Omissions were recorded as an exploratory outcome.

In the *2-back task* (computerized^47^), which assesses working memory, a series of letters is presented (one letter per second), separated by a fixation cross shown for 1 s. Participants had to press the left mouse button if the presented letter was the same as the one two letters before (2-back). After 20 training trials with feedback on their performance, participants completed 80 trials without feedback. The experiment included 24 target letters (12 in the first half and 12 in the second half), with no more than two targets in a row. Our primary outcome measures were the average reaction times as well as the number of correct trials (hits). As exploratory outcomes, we calculated the parameter d-prime (d’), a sensitivity index which relates correct answers to false positives (dʹ = z(hits) − z(false positives))^48^. A higher dʹ indicates a better ability to differentiate the stimuli.

The *instrumental learning task* (ILT; computerized; see e.g.,^49^) measures learning through gains and losses. Participants saw two symbols on the screen: In the gain or loss condition, one of the two symbols was associated to either gaining or losing money, respectively, while the second symbol meant that one’s credit did not change. Participants did not complete any training beforehand, but learned the symbol-outcome associations during the task. Thirty trials per condition (total trials per day = 90) were presented in a random order and the symbol images were randomly matched for each participant to the condition for each session. For each condition, only 80% of the trials were associated with feedback to the participants of either a gain or a loss, and no gain or no loss in the left over 20%. Depending on their symbol choice and the 80/20 feedback contingency, they either won 50 cents, lost 50 cents or there was no change in their credit. Participants had to pick one of two symbols on each trial. They were instructed to maximize earnings and told they would receive the won money as a bonus on top of their monetary compensation. As outcomes, we measured the number of correct responses (hits) and the average reaction time for correct choices (no matter in which trial). Analysis of the instrumental learning task using computational modelling (e.g. the learning rate) will be reported elsewhere.

The *attention and concentration task* (d2-R; paper–pencil test^50^) assesses attention and concentration, accuracy and reaction time during the discrimination of visual stimuli. The participants’ task was to mark a target stimulus (the letter d with a total of two lines either above or below the letter) in a series of the letters d and p that each contain 1–4 lines above or below the respective letter. Participants have 20 s to complete one line, after which correct, incorrect and missed items are counted. Outcome measures included the number of correctly marked target objects (CTO; completed target objects; an index for speed, i.e., how many items were completed in total), the percentage of errors relative to all marked objects (E%; error percent; an index for precision in %), and concentration where the number of errors is subtracted from the number of correctly marked objects (CP; concentration performance; a performance index).

#### Subjective measures

After each of the tasks described above, participants were asked to rate their cognitive performance using computerized *visual analogue scales* from 0 to 100 on two dimensions: effort (Baseline and Test; “Please indicate how effortful this task was personally for you today”, with the anchors ‘not at all effortful’ and ‘very effortful’), and satisfaction with one’s own performance (Baseline and Test; “Please indicate how satisfied you are with your personal performance in the task today” with the anchors ‘very dissatisfied’ and ‘very satisfied’). At the Test session, participants additionally rated their perceived change relative to the Baseline assessment (“Please evaluate how you performed in this task today compared to the last session”, with the anchors ‘a lot worse’ and ‘a lot better’). These ratings were also included as primary outcomes.

Subsequently, we administered five standardized questionnaires, which were preregistered as secondary outcomes, and measured different psychological parameters at both time points: The *State-Trait-Anxiety-Depression-Inventory* (STADI^51^) assesses symptoms of anxiety and depression. It encompasses 40 items (half assessing traits, the other half assessing states, with 4 subscales of 5 items each: emotionality, worry, dysthymia, and anhedonia) that are rated on a 4-point Likert scale from ‘not at all’ to ‘a lot’. The outcomes are a global score and separate scores for depression and anxiety. The trait version was only completed at Baseline. The *Profile of Mood States* (POMS^52^) assesses negative mood with 65 adjectives across the four subscales depressiveness, tiredness, drive, and sullenness. Each adjective is rated on a 5-point Likert scale from ‘not at all’ to ‘very’. The short version of the *Perceived Stress Questionnaire* (PSQ20^53^) assesses the perceived burden/stress in the last 7 days with 20 items across the four subscales worries, tension, joy, and demands. Answers are given on a 4-point Likert scale from ‘almost never’ to ‘most of the time’. Outcome variables were average internal (worries, tension, joy) and external (demands) stressors as well as an average global score. High values indicate high levels of stress (except for the subscale joy, for which the scale is inverted). The *Pittsburgh Sleep Quality Index* (PSQI^54^) assesses quality of sleep. It consists of 18 items that are divided into seven components and are each rated on a 4-point Likert scale. It assesses the occurrence and frequency of sleep-disturbing events over the last three weeks (e.g., times of falling asleep and getting up, hours slept per night, occurrences of sleeping badly, sleep quality, etc.). The *Generic Assessment of Side Effects* (GASE^55^) measures experienced side effects. It includes 36 items, participants score the occurrence and intensity of physical symptoms separately for different body parts on a 4-point Likert scale from ‘0—not present’ to ‘3—strongly’. Participants also have to indicate for each symptom whether they believe it is related to the placebo intake in this study (yes/no).

Lastly, at the Test session, participants were asked to indicate their satisfaction with their group allocation. The question ‘Are you satisfied with your group allocation (placebo or control group)?’ was answered on a VAS from 0 to 100 with the anchors ‘no, not at all’ and ‘yes, absolutely’. They were also asked in a yes-or-no question, whether they would have preferred to be in the other group. The OLP group was then asked a few questions about their adherence to the placebo intake (how regularly they took the placebo capsules, whether they thought the placebo intake improved aspects such as concentration, “mental freshness”, mood, well-being, sleep, and bodily complaints, whether they would recommend the OLP intake to friends or family and whether they would like to continue the OLP intake; each on a VAS from 0 to 100 with the anchors ‘no, not at all’ and ‘yes, very’).

### Data acquisition and analysis

Computerized tasks were administered in a random order using Presentation (version 18.0, Neurobehavioral Systems, Inc. Berkeley, CA, USA), except the learning task which was always administered last. Questionnaires were administered via LimeSurvey (version 4.3.15^56^. All data were processed and analyzed using R in RStudio (RStudio version 2022.07.0, build 548; R version 4.1.3^29^) and JASP (version 0.16.3^57^). Due to the technical problems mentioned above and resulting missing data in some measures, we transparently report the sample sizes of each group for each measure in all Supplementary Tables.

In order to test whether the two groups differed in any of the measures prior to the treatment phase, we compared their task and questionnaires scores at Baseline. To this end, we computed Welch’s two-sample *t*-tests or Pearson’s Chi-squared tests with Yates’ continuity correction for each of our single-time measures at the Baseline. To investigate the effect of OLP intake, we then computed ANOVAs for all measures assessed twice including the between-subject factor *group* (OLP, CTR) and the within-subject factor *time point* (Baseline, Test). The Stroop task analysis of reaction times and accuracy rates included the additional within-subject factor *congruency* (congruent, incongruent). The dual task analysis of reaction times and hit rates also included the within-subjects factor *target* (auditory, visual). Finally, we calculated the amount of complaints/side effects reported in both groups over the course of the study at the Test session and compared the subjective improvement from Baseline to Test at each task between groups, again using Welch’s two-sample *t*-tests.

For all tests, an initial alpha error probability of *p* = 0.05 was assumed, which was further corrected for multiple comparisons (i.e., to *p* = 0.05/14 = 0.004 and *p* = 0.05/15 = 0.003, for 14 objective and 15 subjective tests, respectively). As we report non-significant findings, it has been pointed out that an additional Bayesian analysis framework may complement the standard frequentist approach by providing relative evidence for the null vs. the alternative hypothesis^58,59^. We therefore also report results from the complementary Bayesian *t*-tests and ANOVAs. We employed standard priors of 0.5 for fixed and 1 for random effects as the effect sizes, as implemented in JASP^57^. Bayesian tests produce a Bayes Factor (BF) comparing the relative evidence between the alternative vs. the null hypothesis^60^. A BF_01_ < 3 has been suggested to indicate anecdotal evidence, a BF_01_ > 3 substantial evidence, and BF_01_ > 30 strong evidence for the null compared to the alternative hypothesis^61^. In other words, the larger the BF_01_, the more evidence for the null vs. the alternative hypothesis. The opposite, i.e., evidence for the alternative vs. the null hypothesis (BF_10_), can be computed as BF_10_ = 1/BF_01_ again, with larger BF_10_ indicating more evidence for the alternative compared to the null hypothesis. As those tests may, however, also be sensitive to sample size, especially anecdotal evidence should be treated with caution.

Following the reviewer’s comments, we exploratorily re-ran our primary analyses with gender as a factor and report those results in the Supplement (Tables S12–S16 for objective and Tables S17–S19 for subjective outcomes). However, these results should be interpreted with caution, as our study was not powered to investigate these effects.

## Results

### Baseline group comparison and manipulation checks

All Baseline group differences and respective statistics are reported in Table S2. Overall, between-group Baseline comparisons indicated that the randomization to group allocation resulted in comparable groups. The groups differed significantly in their worry subscale of the STADI, however the corresponding Bayes Factor only indicated anecdotal evidence for a higher worry in the control vs. the OLP group. Furthermore, we observed a non-significant trend towards higher emotionality (*p* = 0.074), behavioral drive (*p* = 0.094), and conscientiousness (*p* = 0.089) in the control group. Importantly, the groups did not differ in any of the other parameters: intelligence, personality traits for emotionality, anhedonia and dysthymia, beliefs about medicine, general treatment expectations, inhibition/approach behavior, and the BIG-5 personality factors neuroticism, extraversion, openness, and agreeableness (all *p*’s > 0.118) at Baseline. This was mirrored by the corresponding Bayes Factors, which indicated some anecdotal, but mostly substantial evidence for the null vs. the alternative hypothesis.

Participants in the OLP group indicated taking the OLPs regularly (93.37 ± 7.34 (*M* ± *SD*) on a scale from 0 ‘not at all’ to 100 ‘very’). Participants in the OLP group were also significantly less satisfied with their group allocation (*M*_OLP_ ± *SD* = 10.90 ± 30.61; *M*_CTR_ ± *SD* = 29.32 ± 28.77; scale 0–100 from ‘no, not at all’ to ‘yes, very’; *t*(75.99) = 3.10, *p* = 0.003) and would have preferred to be in the other group (21 OLP vs. 7 CTR participants, *X*^*2*^(1) = 5.16, *p* = 0.023). The groups did not differ in their amount of reported negative side effects between the two time points (*M*_OLP_ ± *SD* = 4.40 ± 4.25; *M*_CTR_ ± *SD* = 4.26 ± 5.20; scale 0–100 from ‘no, not at all’ to ‘yes, very’; *X*^*2*^(15) = 15.06, *p* = 0.447).

### Effects of OLP intake on objective task measures

**Figure 2:**
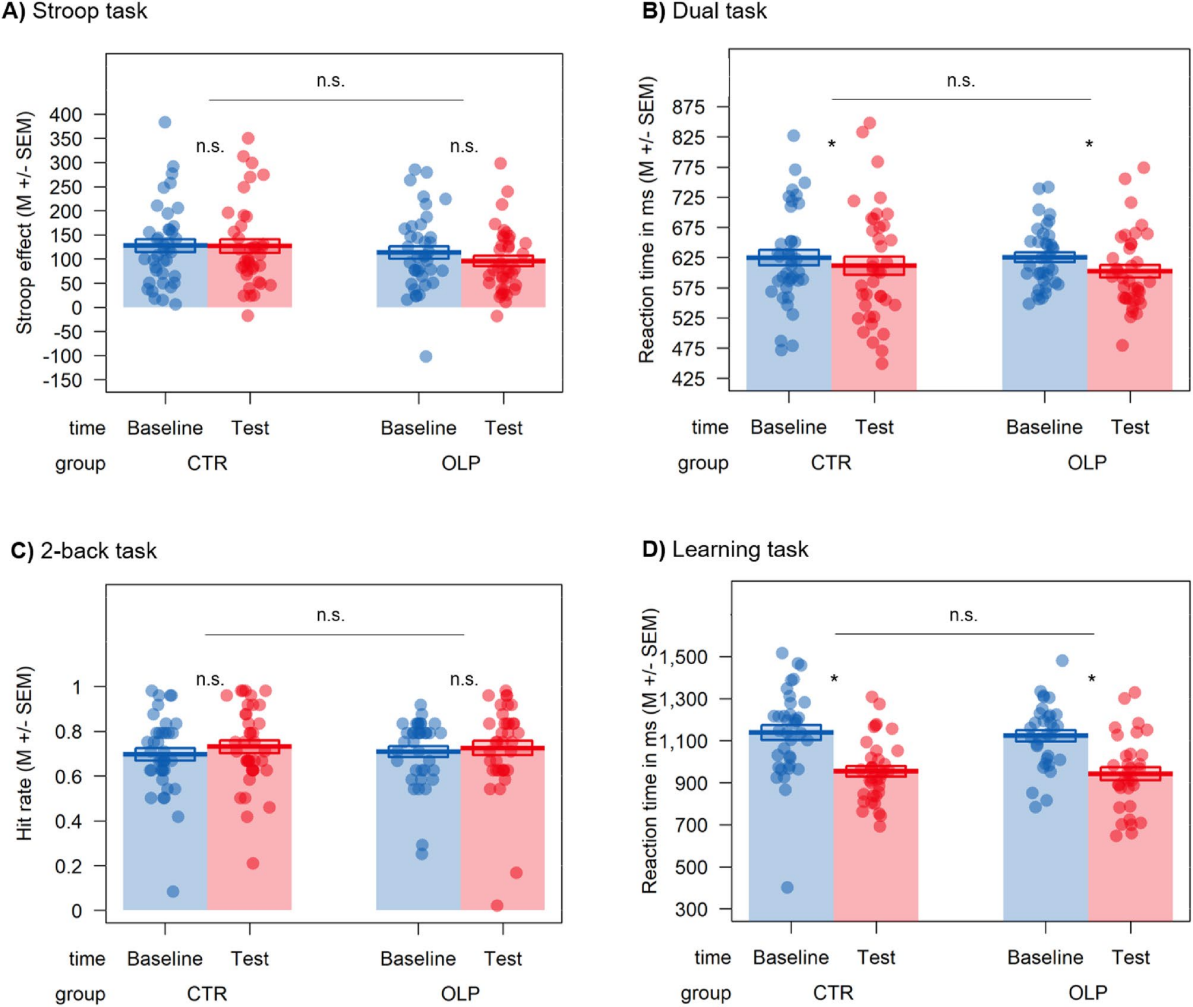
Evidence for training effects, but evidence of absence for any OLP effects on exemplary primary objective outcome measures: (**A**) Stroop task, (**B**) dual task, (**C**) 2-back task, and (**D**) instrumental learning task. The Stroop effect was calculated as the difference in average reaction time between incongruent and congruent trials; Displayed effects for the dual task are averaged over target; Horizontal lines indicate both main effects of group and group × time interactions; *p < 0.05; *n.s.* not significant, *OLP* open-label placebo group, *CTR* control group.

**Figure 3:**
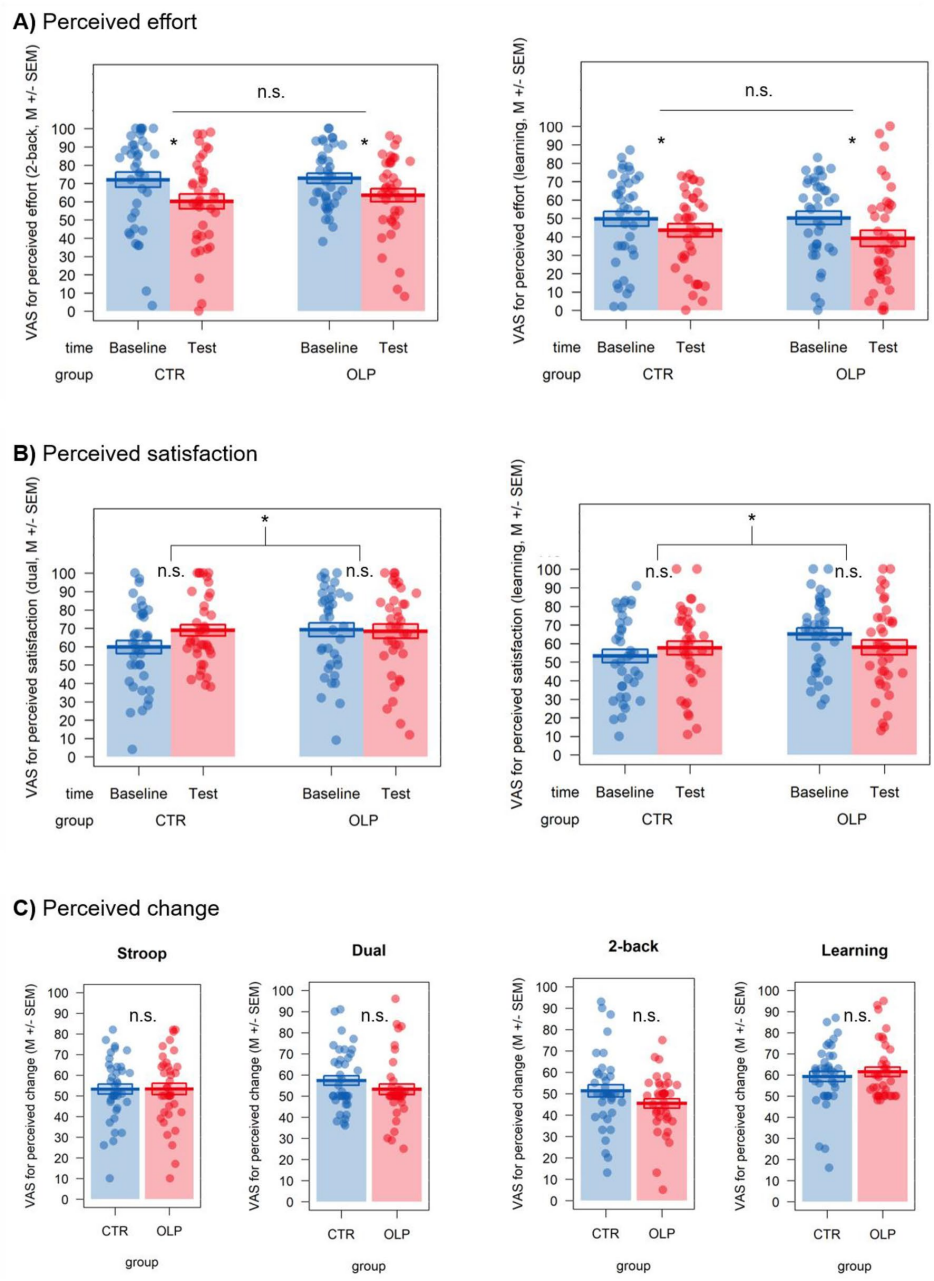
Evidence for time effects, but evidence of absence for group differences in exemplary primary subjective outcome measures regarding (**A**) perceived effort, (**B**) perceived satisfaction, and (**C**) perceived change in one’s performance; Higher values indicate increased effort/satisfaction/change; Horizontal lines indicate main effects of group, bracketed lines indicate group × time interactions; *VAS* visual analogue scale; *p < 0.05; *n.s.* not significant, *OLP* open-label placebo group, *CTR* control group.

First, we analyzed participants’ performance in the objective performance measures (Stroop, dual, 2-back, instrumental learning, and d2). Figures 2 and 3 show exemplary results of objective and subjective outcomes, respectively, as non-displayed outcomes are non-significant.

Contrary to our expectations, none of our primary objective outcome measures demonstrated any group effects (main effects or interactions; all *p*’s > 0.136, ${\upeta }^{2}$’s < 0.001 to 0.03, all BF_01_’s > 1.52), indicating no effect of OLP intake on task performance on our primary outcomes (see Fig. 2A–D, Tables S3–S7). As expected, some tasks showed significant main effects of time, indicating training effects (e.g., dual, learning or d2; see Fig. 2B,D) or replicated commonly reported performance differences within tasks (e.g. congruency effect in the Stroop task, *p* < 0.001, ${\upeta }^{2}$ = 0.08).

### Effects of OLP intake on subjective task measures

Next, we analyzed participants’ subjective evaluation of their objective performance, namely effort invested, satisfaction with their performance, and perceived change from Baseline to Test.

Regarding effort, we again found no effect of OLP intake on these outcomes and Bayesian analyses supported the null over the alternative hypothesis with anecdotal to substantial evidence (main effects of or interactions with group; all *p*’s > 0.176, ${\upeta }^{2}$’s < 0.001 to 0.03; see Fig. 3A). However, we observed main effects of time in two tasks, indicating a decrease in perceived effort in the Test session (d2 and 2-back; all *p*’s < 0.024, ${\upeta }^{2}$’s = 0.08 and 0.06, respectively; see Table S8).

Regarding participants’ subjective assessment of their satisfaction with their own performance, the OLP group reported higher overall satisfaction, independent of time point in the d2 task as indicated by a main effect of group (*p* = 0.039 uncorrected, ${\upeta }^{2}$ = 003; note, however, that this effect was not significant when correcting for multiple comparisons and showed only anecdotal evidence for such an effect in the Bayesian analyses; BF_01_ = 0.74; see Table S9). Furthermore, we observed a group × time interaction in the dual task (*p* = 0.046, ${\upeta }^{2}$ = 0.01; see Fig. 3B left panel; again, this effect did not survive multiple comparison correction). The CTR group showed a significant increase in satisfaction with their performance in the dual task from Baseline to Test (*M*_*diff*_ =  − 9.13), while satisfaction slightly decreased in the OLP group (*M*_*diff*_ = 0.26). However, this effect was only at trend level in the post hoc comparisons (*p* = 0.06; holm-adjusted) and the Bayesian analyses showed anecdotal evidence for the alternative vs. the null hypothesis (BF_10_ = 0.63). Lastly, there was also a significant group × time interaction for the learning task (*p* = 0.050; see Fig. 3B right panel; not significant after multiple comparison correction). Again, none of the post hoc comparisons reached significance (holm-adjusted). Apart from these effects, we observed significant increases in satisfaction across both groups from Baseline to Test in the 2-back task (*p* = 0.002; ${\upeta }^{2}$ = 0.05; see Table S9).

Regarding participants’ subjective assessment of whether their cognitive performance changed in any of the objective tasks from Baseline to Test, we found no significant group effects or other differences and anecdotal (dual, 2-back), but mostly substantial (d2, Stroop, learning) evidence for the null compared to the alternative hypothesis regarding (all *p*’s > 0.108, ${\upeta }^{2}$’s < 0.001 to 0.03, all BF_01_’s > 1.35; see Fig. S10, Fig. 3C).

### Effects of OLP intake on subjective state questionnaires

Lastly, we analyzed the state questionnaires on anxiety, depression, mood, stress, well-being, sleep, and movement/sport activity, which participants had completed before and after OLP intervention. Mirroring the above analyses, we found no OLP effects on any of these outcomes (see Table S11). However, we found that motivation and joy decreased while fatigue increased across both groups from Baseline to Test (*p*’s < 0.033).

In a post-experimental questionnaire at Test, participants had a low belief in OLP induced performance improvement in the computer tasks (*M* ± *SD* = 12.37 ± 18.15) and their performance in everyday life (15.97 ± 23.91) after the intake period. We also found low subjective scores (on 0–100 scales from ‘no, not at all’ to ‘yes, very’) for an improvement in concentration (15.20 ± 20.71), “mental freshness” (15.67 ± 22.50), mood (12.52 ± 19.85), well-being (13.77 ± 21.17), sleep (10.72 ± 17.43), and physical issues (7.90 ± 14.57) through OLP intake. Participants would rather not recommend OLPs to friends or family (29.45 ± 25.28, scale 0–100 from ‘no, not at all’ to ‘yes, very’) and would not like to continue the intake (26.97 ± 25.15, scale 0–100 from ‘no, not at all’ to ‘yes, very’).

## Discussion

Here, we investigated the performance-enhancing effects of OLPs using an extensive battery of subjective and objective cognitive performance measures and well-being. Our analyses suggest that this treatment had no significant impact on any of the outcomes, compared to a no-treatment control condition. As expected, participants’ performance generally improved from Baseline to Test in most tasks, most likely reflecting training effects in tasks assessing attention and concentration, working memory, and instrumental learning^62,63^. We also replicated known performance differences within a task, such as learning effects over time and a difference in reaction time and accuracy between congruent and incongruent trials in the Stroop task^45^, which underscores the validity of our employed tasks. These learning effects in objective measures were accompanied by the perception of decreased effort in and increased satisfaction with one’s task performance in some of these tasks. However, performance improvement from Baseline to Test was not enhanced by the 3-week OLP treatment—neither on the objective nor on the subjective level. This was also confirmed by Bayesian analyses, which is reassuring considering our smaller-than-planned sample size. In the following we discuss the absence of findings and possible reasons for this, including limitations of the present study.

The lack of objective improvements in cognitive performance is not unusual. Previous studies using deceptive placebos also yielded mixed results for objective parameters in healthy participants^10,12,64^. For example, Blokland^64^ found no effect of deceptive placebos on performance regarding word learning, working memory, the Tower of London task, or spatial pattern separation, which is in line with our findings. Similarly, an OLP application failed to improve cognitive performance in medical students where exam results served as a proxy for objective cognitive performance^24^.

The absence of any improvement in subjective outcome including perceived performance and satisfaction therewith as well as no improvements of general well-being, however, is surprising given that many studies in clinical, but also non-clinical samples report subjective improvement under OLP treatment. Importantly, this also applies to trials with no changes in objective measures, which indicates that the perception of the patient or healthy participant can deviate from measurable outcome. In contrast to objective outcome parameters which are judged against an external criterion, subjective outcomes require the individual to compare any perceived changes in the relevant metric (e.g., processing speed in a cognitive task) to an internal standard to decide whether the change is meaningful, i.e., significantly different from noise. This threshold is likely to be influenced by the individual’s motivation to categorize changes as meaningful—particularly when they are subtle in nature. A strong motivation will lower the threshold whereas a weak motivation will lead to a higher threshold. Furthermore, the change must be attributed to the treatment (as opposed to coincidence). Several of our subjective outcome measures seem to indicate a rather critical assessment of their treatment in the OLP group which could reflect a high threshold to detect change. Participants in this group were significantly less satisfied with their group allocation than the control group, would have preferred to be in the control group, would not recommend OLPs to others and provided low ratings on their willingness to continue the treatment. Moreover, 5% of participants were excluded because they indicated that they had taken the OLP less than 70% of the time and 12% did not attend the second session.

Psychological theories consider motivation or attitude towards an entity as the product of two components—expectancy and value^65^. While expectancy refers to the perceived likelihood that a certain action will lead to a desired outcome, value indicates the weight assigned to the outcome when assessing its significance or subjective importance. Applied to the context of OLP treatment, an individual’s motivation to detect change would be defined by their expectation that the OLP will lead to improved task performance (expectancy) and the desire to experience such an effect (value). We posit that both factors might have been rather low in our sample and therefore led non-significant subjective improvements. In clinical populations, the value of a potential treatment success is undoubtedly high. Chronic diseases often have severe consequences for the patient in all facets of their life. This disease burden creates a strong desire for relief, which is known to be a strong predictor of treatment outcomes^66^, including OLP^67–69^. In healthy individuals, the value of treatment success might be less clear and depend on the context and envisaged outcome. While the prospect of better task performance in an exam might be very valuable, performance improvement in an experimental task commonly has no implications for the individual beyond the experiment and hence only little value. This difference in value might therefore explain why OLP improve subjective outcome parameters such as distress and well-being in healthy medical students in an exam situation^24^ but had no effect in our experimental study.

Expectancy as the second determinant has been a focus of several investigations into factors determining placebo outcome in the past. For deceptive placebo treatments, beneficial effects are thought to depend on an individual’s positive treatment expectation^70^. Whether strong positive expectations are as critical for OLP treatment as for deceptive placebo treatments remains to be investigated^16,24,64,71–74^. El Brihi et al.^75^ reported that positive expectations predicted the positive influence of OLP treatment on well-being and physical symptom reduction (e.g., headache, dizziness, diarrhea) in healthy undergraduates. Expectations regarding the effect of OLP treatment are likely to reflect the general attitudes of an individual to this type of treatment. A recent survey of 532 respondents we conducted revealed that people were generally open towards taking placebos, but more when it would be taken as an add-on. This survey study also indicated that research studies play the biggest part in convincing people to try placebos^76^. An earlier study also showed that people seem to value the honesty and transparency in OLP treatments^77^. However, another study^78^ showed that acceptance of OLP treatment is lower than for deceptive treatment, which was linked to lower expectations for open placebo application. Similarly, a recent study using qualitative methods in young healthy individuals confirmed that openly prescribed placebos were seen as less powerful than deceptive ones^78^. These findings indicate that while the transparency of open-label application of placebo may be welcomed, it may negatively impact the judgement of potential treatment effects.

Explicit verbal information about the nature and potential benefits of placebos are a key component of many OLP studies^16^ and are intended to maximize expectations irrespective of the general attitude towards OLP treatment at study entry. Treatment rationale during OLP administration is itself an important area of research and may be crucial for treatment success^78–80^. In the current study, we stayed relatively neutral in our patient information and highlighted that we aimed to explore whether OLP treatment can modulate cognitive performance, leaving open the possibility that it might have no effect at all (see Table S1 in the Supplement). These more ‘modest’ instructions may have decreased participants’ expectations and thereby limited the OLP effect on treatment outcomes^18^. The two groups showed no difference in treatment expectations or general beliefs about medication prior to group allocation, which speaks against the idea that the OLP group might have been more skeptical of this kind of treatment from the outset. Instead, this finding points towards a change in this group that occurs during the treatment. Extensions of the original expectancy × value theory propose that costs can be a third factor (in addition to value and expectancy) determining motivation. In drug trials, costs commonly relate to side effects, which can affect patients’ overall satisfaction with the treatment but can also have a direct negative impact on treatment outcome. However, side effects were not more common in the OLP group than the control group suggesting that they played no major role in cost estimations in the OLP group. While this low overall assessment of the OLP treatment could indicate dissatisfaction with treatment outcome, it could also reflect dissatisfaction with the additional effort the OLP group had to invest by taking the tablets which was not required in the control group. As the act of taking the “medication” is seen as an integral (conditioned) part of the induction of a placebo effect, it could not be added to the control group to control for its impact. Cost considerations (either in the form of side effects or effort invested) should nonetheless be taken into account when planning and interpreting studies investigating effects of OLP treatment.

Our findings have to be interpreted in light of some limitations. First, our sample size was smaller than anticipated. To explore the robustness of our findings, we therefore decided to also apply Bayesian statistics. These analyses provided direct relative evidence for the null vs. the alternative hypothesis. Moreover, to ensure that the results we report are also of clinical significance, we were mainly interested in medium to large effects which can also be detected in a smaller sample such as ours. Second, not all tasks were administered in a randomized order. Especially for the learning task, which was always administered last, we therefore cannot rule out that the results are confounded by an increasing level of fatigue. However, as our findings compare two groups, such an effect (and also training effects) should occur in both groups. Third, we chose established cognitive tasks that had been successfully implemented in previous work and which are sensitive to performance improvement through OLPs while avoiding ceiling effects (e.g., 2-back vs. 3-back). It remains to be investigated whether more cognitively demanding tasks (e.g., 3- or 4-back) would be even more sensitive to the effect of OLPs. Fourth, previous work has shown that a 3-week treatment with OLPs is sufficient to induce effects^24,33–35^. Nevertheless, it would be an exciting future avenue to investigate the optimal duration of OLP intake that maximizes beneficial effects on cognitive performance. In this context, comparing acute vs. long-term effects would be a compelling focus for future work.

Together, our findings do not support the assumption that OLPs can enhance cognitive functioning in healthy adults, and thereby showcase potential limitations of OLP treatments. Instead, we highlight important considerations for future studies aiming to include OLP treatments. Our proposed framework that follows expectancy-value considerations of motivation suggests several trajectories for future research to gain a more comprehensive understanding of the mechanisms and effects of OLP treatments and predictors for an individual’s response. First, the role of expectancy, value and costs, their respective determinants and interactions need to be investigated in more detail. A better understanding could not only help patient stratification but also aid the systematic investigation of differences in OLP responses between clinical and non-clinical groups and between objective and subjective outcome measures. Second, information about individual expectation, value and cost estimations could inform computational models which promise to reveal (neural) mechanisms underlying observable behaviour. Lastly, a deeper understanding could guide efforts to improve OLP outcome. For instance, expectations might be easier to modulate than costs, which are often outside the control of the practitioner. Information provided before OLP treatment could therefore focus on raising expectations while managing costs or may be tailored to fit the needs or preferences of the individual patient.

### Supplementary Information

Supplementary Information.

## Data Availability

The data used for the analysis has been uploaded to the Open Science Framework project (https://osf.io/gzkxy/)^82^.
