# Supplementary material for: Open-label placebo treatment does not enhance cognitive abilities in healthy volunteers
Source: Sci Rep. 2023 Nov 9;13:19468. doi: 10.1038/s41598-023-45979-3 (PMC10636058; doi:10.1038/s41598-023-45979-3)
Supplement: Supplementary file 1 — Supplementary Information. [file 41598_2023_45979_MOESM1_ESM.docx]

Supplement to

**Open-label placebo treatment does not enhance cognitive abilities in healthy volunteers**

**Helena Hartmann^1*^, Katarina Forkmann^1^, Katharina Schmidt^1^,
Julian Kleine-Borgmann^1^, Johanna Albers^1^, Katja Wiech^2^, & Ulrike Bingel^1^**

*^1^Clinical Neurosciences, Department of Neurology and Center for Translational Neuro- and Behavioral Sciences (C-TNBS), University Hospital Essen, Essen, Germany*

*^2^Nuffield Department of Clinical Neurosciences, John Radcliffe Hospital, University of Oxford, Haedington, Oxford, UK*

* Corresponding author: [helena.hartmann@uk-essen.de](mailto:helena.hartmann@uk-essen.de), University Hospital Essen, Department of Neurology, Clinical Neurosciences, Center of Translational Neuro- and Behavioural Sciences, University Medicine Essen, Hufelandstraße 55, 45147 Essen, Germany

Co-author emails and ORCIDs: [helena.hartmann@uk-essen.de](mailto:helena.hartmann@uk-essen.de) (0000-0002-1331-6683), [katarina.forkmann@uk-essen.de](mailto:katarina.forkmann@uk-essen.de) (0000-0002-2219-6935), [katharina.schmidt@uk-essen.de](mailto:katharina.schmidt@uk-essen.de), [julian.kleine-borgmann@uk-essen.de](mailto:julian.kleine-borgmann@uk-essen.de) (0000-0001-7941-0595), [bonjour@johanna-albers.de](mailto:bonjour@johanna-albers.de) (0009-0001-6649-6438), [katja.wiech@ndcn.ox.ac.uk](mailto:katja.wiech@ndcn.ox.ac.uk) (0000-0002-5062-1046), [ulrike.bingel@uk-essen.de](mailto:ulrike.bingel@uk-essen.de) (0000-0002-9528-3204).

## OLP instructions

In Table S1, we report the information participants received about the study itself and/or the OLP intake, both in German and English.

Table S1. Excerpts of participant information regarding the study and OLP intake instructions.

| **Type** | **German (original)** | **English (translated)** |
| --- | --- | --- |
| Flyer texts for social media | „Gesunde Proband/innen für Placebostudie gesucht. Für eine Studie zum Thema „Einfluss von Placebos auf die kognitive Leistungsfähigkeit“ sucht die Klinik für Neurologie gesunde ProbandInnen zwischen 18 und 40 Jahren. In der Studie soll untersucht werden, ob die Einnahme eines Placebos (d.h. einer Tablette ohne Wirkstoff) die kognitive Leistung beeinflussen kann. Während der Studie werden verschiedene kurze Tests am PC ausgeführt. Es finden 2 Termin im UK Essen im Abstand von ca. 3 Wochen statt. In diesen 3 Wochen nimmt die Hälfte der Probanden täglich zwei Placebos ein.“  „Gesunde Teilnehmer gesucht! Wir wollen testen, wie sich eine offene Placebogabe auf kognitive Leistungen auswirkt.“ | "Healthy participants wanted for placebo study. The Department of Neurology is looking for healthy volunteers between the ages of 18 and 40 to participate for a study on the influence of placebos on cognitive performance. The study will examine whether taking a placebo (i.e., a tablet without an active ingredient) can affect cognitive performance. During the study, various short tasks will be performed on the PC. There will be 2 appointments in the UK Essen about 3 weeks apart. During these 3 weeks, half of the participants will take two placebos daily."  "Healthy participants wanted! We want to test how open-label placebo administration affects cognitive performance." |
| Recruitment email | „Für unsere Studie zum Thema Placebo und Kognition am Universitätsklinikum Essen suchen wir noch gesunde Proband(inn)en. (…) In dieser Studie möchten wir untersuchen ob die Einnahme eines Placebos (d.h. einer Tablette ohne Wirkstoff) die kognitive Leistungsfähigkeit beeinflussen kann. Wenn Sie an dieser Studie teilnehmen möchten, wäre der Ablauf folgendermaßen: Sie kommen zu einem ersten Termin, absolvieren die kognitiven Tests und werden entweder der Placebo- oder der Kontrollgruppe zugeteilt. Falls Sie der Placebogruppe zugelost werden, müssen Sie über einen Zeitraum von 3 Wochen zwei mal täglich ein Placebo einnehmen. Falls Sie der Kontrollgruppe zugelost werden, nehmen Sie kein Placebo ein. Sie kommen zu einem zweiten Termin nach ca. 21 Tagen, an dem sie lediglich die Tests absolvieren.“ | "For our study on placebo and cognition at the University Hospital Essen we are still looking for healthy participants. (...) In this study, we would like to investigate whether taking a placebo (i.e., a tablet without an active ingredient) can affect cognitive performance. If you would like to participate in this study, the procedure would be as follows: You will come in for an initial appointment, complete the cognitive tasks, and be assigned to either the placebo or the control group. If you are randomly assigned to the placebo group, you will be required to take a placebo twice a day for a period of 3 weeks. If you are assigned to the control group, you will not take a placebo. You will come in for a second appointment after about 21 days, where you will only complete the tasks." |
| Instructions at the baseline session | „Wir untersuchen in dieser Studie ob ein Placebo Einfluss auf die kognitive Leistungsfähigkeit bei gesunden Probanden und Probandinnen haben kann. (…) Wenn Sie mit den Fragebögen fertig sind, nehmen Sie sich bitte eine der vorbereiteten Schachteln. Diese enthalten entweder ein Placebo oder sind leer. Öffnen Sie die Schachtel bitte erst zu Hause und folgen Sie den darin liegenden Anweisungen.“ | "In this study, we are investigating whether a placebo can have an effect on cognitive performance in healthy participants. (...) When you are finished with the questionnaires, please take one of the prepared boxes. These either contain a placebo or are empty. Please do not open the box until you get home and follow the instructions inside." |
| Instructions at the test session | „Denken Sie bitte daran, den Zettel den Sie in Ihrer Box hatten in diese Box hier zu legen. Wichtig: Ich darf auch heute noch nicht wissen, welcher Gruppe sie zugordnet waren. Ich bitte Sie daher – auch untereinander (bei zwei ProbandInnen) nicht über die Zuteilung oder Einnahme zu sprechen.“ | "Please remember to put the piece of paper you had in your box in this box here. Important: I cannot know even today which group you were assigned to. I therefore ask you - even among yourselves (for two participants) – not to talk about the assignment or intake." |

## Baseline group differences

Table S2 depicts the baseline demographics, measures and differences between groups.

Table S2. Overview of baseline group differences for questionnaires.

| Measure & Subscale | *Mean (SD) or frequency (n)* | | *n* | *t/Chi^2^* | *df* | *p* | BF_01_ |
| --- | --- | --- | --- | --- | --- | --- | --- |
|  | OLP | CTR | OLP/CTR |  |  |  |  |
| Age | 28.10 (10.46) | 28.82 (6.93) | 40/38 | 0.36 | 68.05 | .721 | --- |
| Gender  Female  Male | 22  18 | 23  15 | 40/38 | 0.07 | 1 | .791 | --- |
| Education  GSCE O-level  GSCE A-level  University level | 0  25  15 | 3  21  14 | 40/38 | 3.33 | 2 | .189 | --- |
| MWT-B | 28.47 (3.88) | 28.57 (3.14) | 40/38 | -0.13 | 71.25 | .900 | 4.22 |
| STADI (trait)  Emotionality  Worry  Anhedonia  Dysthymia | 2.21 (0.26)  2.11 (0.25)  2.49 (0.19)  2.62 (0.23) | 2.32 (0.28)  2.21 (0.18)  2.56 (0.24)  2.68 (0.24) | 40/38  40/38  40/38  40/38 | 1.81  2.04  1.58  1.09 | 74.74  70.62  73.41  75.36 | .074  **.046**  .118  .281 | 1.04  0.74  1.47  2.55 |
| BMQ  General overuse  General harm  General benefit  Sensitive soma. | 13.73 (3.21)  9.23 (2.75)  13.73 (3.21)  9.33 (3.58) | 14.08 (3.20)  8.97 (2.90)  14.08 (3.20)  9.18 (3.71) | 40/38  40/38  40/38  40/38 | 0.49  -0.39  0.49  -0.17 | 75.82  75.18  75.82  75.40 | .627  .696  .627  .865 | 3.85  4.65  3.85  4.20 |
| TEX-Q  Symptom impr.  Positive effect  Negative effect  Side effects  Process  Behav. control  Total | 2.71 (2.03)  2.03 (1.74)  0.36 (0.73)  0.86 (0.92)  5.40 (3.27)  5.10 (2.86)  2.48 (1.14) | 2.96 (1.85)  1.84 (1.19)  0.26 (0.85)  0.79 (0.90)  5.32 (3.10)  5.13 (2.27)  2.48 (0.94) | 40/38  40/38  40/38  40/38  40/38  40/38  40/38 | 0.57  -0.55  -0.55  -0.33  -0.12  0.05  -0.01 | 75.89  69.27  73.08  75.91  76.00  73.65  74.56 | .574  .587  .584  .739  .907  .957  .990 | 3.70  3.75  3.73  4.05  4.24  4.26  4.26 |
| BIS/BAS  BIS  BAS drive  BAS reward  BAS fun | 19.28 (2.59)  11.68 (2.43)  16.33 (1.70)  11.78 (1.80) | 18.74 (2.77)  12.58 (2.29)  16.61 (1.70)  12.21 (1.99) | 40/38  40/38  40/38  40/38 | -0.89  1.69  0.73  1.01 | 74.97  75.99  75.79  74.36 | .379  .094  .469  .315 | 3.03  1.25  3.38  2.72 |
| BFI  Neuroticism  Extraversion  Openness  Conscientiousness  Agreeableness | 2.83 (0.51)  3.09 (0.56)  3.20 (0.65)  3.36 (0.64)  3.19 (0.65) | 2.92 (0.62)  3.20 (0.54)  3.17 (0.62)  3.61 (0.61)  3.22 (0.78) | 40/38  40/38  40/38  40/38  40/38 | 0.74  0.88  -0.20  1.72  0.22 | 71.89  76.00  76.00  76.00  72.12 | .460  .382  .841  .089  .824 | 3.34  3.05  4.18  1.20  4.17 |
| *Note.* SD = standard deviation; OLP = open-label placebo; GSCE = General Certificate of Education; CTR = control; BF_01_ = Bayes Factor evidence for the null vs. the alternative hypothesis; MWT-B = Mehrfachwahl-Wortschatz-Intelligenztest [Multiple Choice Vocabulary Intelligence Test]; STADI = State-Trait-Anxiety-Depression-Inventory; BMQ = Beliefs About Medicines Questionnaire; TEX-Q = Treatment Expectation Questionnaire; BIS/BAS = Behavioral Inhibition/Behavioral Approach System Questionnaire; BFI = Big Five Inventory. | | | | | | | |

## Objective task outcomes

Tables S3-7 show the analyses of the objective tasks. In the Stroop task, participants across both groups were faster and more accurate in congruent than incongruent trials as indicated by main effects of congruency, both for average reaction times and hit rates (*p’s* < .001; see Table S3). In the dual task, participants across both groups were faster but also had less hits/more omissions for auditory vs. visual targets and generally became faster from baseline to test as indicated by main effects of target for all three outcome measures and a main effect of time for RT only (all *p’s* < .009; see Table S4). In the 2-back task, participants across both groups showed greater sensitivity/discriminability at test vs. baseline (main effect of time for d’; *p* = .035; see Table S5). In the instrumental learning task, participants across both groups were faster in choosing a symbol at test than at baseline (main effect of time for the RT data; *p* < .001; see Table S6). Finally, in the d2 task, participants across both groups significantly increased their number of finished target objects, reduced their error percentage, and concentration from baseline to test (significant main effects of time for all three outcome parameters; all *p*’s < .001; see Table S7). Bayesian analyses confirmed these effects, with very strong evidence for the alternative vs. the null hypothesis.

Table S3. Mixed ANOVAs and BF_01_’s for the three outcomes of the Stroop task.

| Measure & Effect | | $SS$ | | *df*‘s | | Mean Square | | $F$ | | $p$ | | $ƞ^{2}$ | | BF_01_ |
| --- | --- | --- | --- | --- | --- | --- | --- | --- | --- | --- | --- | --- | --- | --- |
| Reaction time |  |  |  |  |  |  |  |  |  |  |  |  |  |  |
| Time |  | 18850.12 |  | 1,74 |  | 18850.122 |  | 2.059 |  | .156 |  | 0.001 |  | 2.603 |
| Group |  | 320475.34 |  | 1,74 |  | 320475.344 |  | 2.277 |  | .136 |  | 0.025 |  | 1.896 |
| Congruency |  | 1.013e+6 |  | 1,74 |  | 1.013e+6 |  | 220.671 |  | **< .001** |  | 0.078 |  | < 0.001 |
| Time x Group |  | 6.71 |  | 1,74 |  | 6.710 |  | 7.329e-4 |  | .978 |  | < 0.001 |  | 4.055 |
| C x T |  | 1328.57 |  | 1,74 |  | 1328.571 |  | 0.710 |  | .402 |  | < 0.001 |  | 5.220 |
| C x G |  | 9082.64 |  | 1,74 |  | 9082.642 |  | 1.978 |  | .164 |  | < 0.001 |  | 1.518 |
| C x T x G |  | 1595.89 |  | 1,74 |  | 1595.888 |  | 0.853 |  | .359 |  | < 0.001 |  | 27.185 |
| Accuracy |  |  |  |  |  |  |  |  |  |  |  |  |  |  |
| Time |  | < 0.01 |  | 1,75 |  | 0.004 |  | 0.614 |  | .436 |  | 0.001 |  | 8.592 |
| Group |  | < 0.01 |  | 1,75 |  | 0.004 |  | 0.305 |  | .583 |  | 0.001 |  | 10.450 |
| Congruency |  | 0.15 |  | 1,75 |  | 0.147 |  | 16.306 |  | **< .001** |  | 0.054 |  | 0.015 |
| Time x Group |  | 0.01 |  | 1,75 |  | 0.007 |  | 1.186 |  | .280 |  | 0.003 |  | 35.047 |
| C x T |  | 0.01 |  | 1,75 |  | 0.006 |  | 0.929 |  | .338 |  | 0.002 |  | 8.732 |
| C x G |  | < 0.01 |  | 1,75 |  | < 0.001 |  | 0.055 |  | .815 |  | < 0.001 |  | 11.367 |
| C x T x G |  | 0.01 |  | 1,75 |  | 0.008 |  | 1.316 |  | .255 |  | 0.003 |  | 194.591 |
| Stroop effect |  |  |  |  |  |  |  |  |  |  |  |  |  |  |
| Time |  | 2657.14 |  | 1,74 |  | 2657.143 |  | 0.710 |  | .403 |  | 0.003 |  | 5.709 |
| Group |  | 8043.52 |  | 1,74 |  | 8043.524 |  | 0.863 |  | .356 |  | 0.008 |  | 3.719 |
| Time x Group |  | 2288.13 |  | 1,74 |  | 2288.129 |  | 0.610 |  | .437 |  | 0.002 |  | 13.546 |
| *Note.* Type III Sum of Squares (SS); Sample sizes were *n* = 38 for the open-label-placebo group and *n* = 38/39 for the control group; BF_01_ = evidence for the null vs. the alternative hypothesis; C = congruency; T = time; G = group. The alpha error probability corrected for multiple comparisons in 14 objective tests was *p* = .05/14 = .004. | | | | | | | | | | | | | | |

Table S4. Mixed ANOVAs and BF_01_’s for the three outcomes of the dual task.

| Measure & Effect | | $SS$ | | *df*‘s | | Mean Square | | $F$ | | $p$ | | $ƞ^{2}$ | | BF_01_ |
| --- | --- | --- | --- | --- | --- | --- | --- | --- | --- | --- | --- | --- | --- | --- |
| Reaction time |  |  |  |  |  |  |  |  |  |  |  |  |  |  |
| Time |  | 34480.07 |  | 1,74 |  | 34480.073 |  | 13.579 |  | **< .001** |  | 0.011 |  | 0.075 |
| Group |  | 9.06 |  | 1,74 |  | 9.059 |  | 6.368e-4 |  | .980 |  | < 0.001 |  | 6.247 |
| Target |  | 1.477e+6 |  | 1,74 |  | 1.477e+6 |  | 320.960 |  | **< .001** |  | 0.453 |  | < .001 |
| Time x Group |  | 1200.58 |  | 1,74 |  | 1200.583 |  | 0.473 |  | .494 |  | 0.003 |  | 7.066 |
| Ta x Ti |  | 682.70 |  | 1,74 |  | 682.700 |  | 0.308 |  | .580 |  | 0.002 |  | 2.296 |
| Ta x G |  | 1215.70 |  | 1,74 |  | 1215.697 |  | 0.264 |  | .609 |  | 0.003 |  | 8.079 |
| Ta x Ti x G |  | 64.34 |  | 1,74 |  | 64.337 |  | 0.029 |  | .865 |  | < 0.001 |  | 111.427 |
| Hits |  |  |  |  |  |  |  |  |  |  |  |  |  |  |
| Time |  | 15.30 |  | 1,75 |  | 15.304 |  | 2.742 |  | .102 |  | 0.012 |  | 2.366 |
| Group |  | 0.14 |  | 1,75 |  | 0.136 |  | 0.016 |  | .901 |  | 0.001 |  | 3.487 |
| Target |  | 28.50 |  | 1,75 |  | 28.503 |  | 14.287 |  | **< .001** |  | 0.021 |  | 0.023 |
| Time x Group |  | 8.41 |  | 1,75 |  | 8.408 |  | 1.506 |  | .224 |  | 0.006 |  | 4.071 |
| Ta x Ti |  | 0.01 |  | 1,75 |  | 0.011 |  | 0.015 |  | .901 |  | < .001 |  | 5.828 |
| Ta x G |  | 1.69 |  | 1,75 |  | 1.685 |  | 0.845 |  | .361 |  | 0.001 |  | 3.416 |
| Ta x Ti x G |  | 0.47 |  | 1,75 |  | 0.466 |  | 0.652 |  | .422 |  | 0.003 |  | 36.871 |
| Misses |  |  |  |  |  |  |  |  |  |  |  |  |  |  |
| Time |  | 15.29 |  | 1,75 |  | 15.292 |  | 2.837 |  | .096 |  | 0.012 |  | 2.194 |
| Group |  | 0.84 |  | 1,75 |  | 0.837 |  | 0.101 |  | .752 |  | 0.006 |  | 4.288 |
| Target |  | 12.41 |  | 1,75 |  | 12.410 |  | 7.274 |  | **.009** |  | 0.010 |  | 0.478 |
| Time x Group |  | 7.76 |  | 1,75 |  | 7.760 |  | 1.440 |  | .234 |  | 0.006 |  | 3.257 |
| Ta x Ti |  | 0.01 |  | 1,75 |  | 0.011 |  | 0.016 |  | .899 |  | < .001 |  | 5.304 |
| Ta x G |  | 0.57 |  | 1,75 |  | 0.565 |  | 0.331 |  | .567 |  | 0.004 |  | 5.750 |
| Ta x Ti x G |  | 0.32 |  | 1,75 |  | 0.323 |  | 0.460 |  | .500 |  | 0.002 |  | 97.158 |
| *Note.* Type III Sum of Squares (SS); Sample sizes were *n* = 37/38 for the open-label-placebo group and *n* = 39 for the control group; BF_01_ = evidence for the null vs. the alternative hypothesis; Ta = Target; Ti = time; G = group. The alpha error probability corrected for multiple comparisons in 14 objective tests was *p* = .05/14 = .004. | | | | | | | | | | | | | | |

*Table S5. Mixed ANOVAs and BF_01_’s for the three outcomes of the 2-back task.*

| Measure & Effect | | $SS$ | | *df*‘s | | Mean Square | | $F$ | | $p$ | | $ƞ^{2}$ | | BF_01_ |
| --- | --- | --- | --- | --- | --- | --- | --- | --- | --- | --- | --- | --- | --- | --- |
| Reaction time |  |  |  |  |  |  |  |  |  |  |  |  |  |  |
| Time |  | 1685.59 |  | 1,73 |  | 1685.588 |  | 0.213 |  | .646 |  | 0.007 |  | 1.811 |
| Group |  | 3156.30 |  | 1,73 |  | 3156.303 |  | 0.144 |  | .706 |  | 0.001 |  | 3.537 |
| Time x Group |  | 747.78 |  | 1,73 |  | 747.782 |  | 0.094 |  | .759 |  | 0.003 |  | 7.676 |
| Hit rate |  |  |  |  |  |  |  |  |  |  |  |  |  |  |
| Time |  | 0.03 |  | 1,74 |  | 0.025 |  | 3.156 |  | .080 |  | 0.006 |  | 0.587 |
| Group |  | 0.004 |  | 1,74 |  | 0.004 |  | 0.008 |  | .929 |  | < 0.001 |  | 8.422 |
| Time x Group |  | 0.003 |  | 1,74 |  | 0.003 |  | 0.377 |  | .541 |  | 0.006 |  | 15.452 |
| d-prime |  |  |  |  |  |  |  |  |  |  |  |  |  |  |
| Time |  | 1.05 |  | 1,74 |  | 1.045 |  | 4.598 |  | .035 |  | 0.010 |  | 0.980 |
| Group |  | 0.06 |  | 1,74 |  | 0.056 |  | 0.046 |  | .831 |  | 0.005 |  | 3.600 |
| Time x Group |  | 0.11 |  | 1,74 |  | 0.111 |  | 0.489 |  | .486 |  | 0.001 |  | 5.381 |
| *Note.* Type III Sum of Squares (SS); Sample sizes were *n* = 36/37 for the open-label-placebo group and *n* = 39 for the control group; BF_01_ = evidence for the null vs. the alternative hypothesis. The alpha error probability corrected for multiple comparisons in 14 objective tests was *p* = .05/14 = .004. | | | | | | | | | | | | | | |

Table S6. Mixed ANOVAs and BF_01_’s for the two outcomes of the instrumental learning task.

| Measure & Effect | | $SS$ | | *df*‘s | | Mean Square | | $F$ | | $p$ | | $ƞ^{2}$ | | BF_01_ |
| --- | --- | --- | --- | --- | --- | --- | --- | --- | --- | --- | --- | --- | --- | --- |
| Reaction time |  |  |  |  |  |  |  |  |  |  |  |  |  |  |
| Time |  | 1.12 |  | 1,66 |  | 1.124 |  | 97.809 |  | **< .001** |  | 0.217 |  | < 0.001 |
| Group |  | 0.01 |  | 1,66 |  | 0.006 |  | 0.128 |  | .722 |  | 0.001 |  | 3.438 |
| Time x Group |  | 0.001 |  | 1,66 |  | 0.001 |  | 0.012 |  | .914 |  | < 0.001 |  | 4.054 |
| Hits |  |  |  |  |  |  |  |  |  |  |  |  |  |  |
| Time |  | 1643.81 |  | 1,66 |  | 1643.806 |  | 2.993 |  | .088 |  | 0.009 |  | 1.746 |
| Group |  | 513.33 |  | 1,66 |  | 513.334 |  | 0.225 |  | .637 |  | 0.003 |  | 3.541 |
| Time x Group |  | 638.28 |  | 1,66 |  | 638.276 |  | 1.162 |  | .285 |  | 0.003 |  | 5.132 |
| *Note.* Type III Sum of Squares (SS); Sample sizes were *n* = 32 for the open-label-placebo group and *n* = 36 for the control group; BF_01_ = evidence for the null vs. the alternative hypothesis. The alpha error probability corrected for multiple comparisons in 14 objective tests was *p* = .05/14 = .004. | | | | | | | | | | | | | | |

*Table S7. Mixed ANOVAs and BF_01_’s for the three outcomes of the d2 task.*

| Measure & Effect | | $SS$ | | *df*‘s | | Mean Square | | $F$ | | $p$ | | $ƞ^{2}$ | | BF_01_ |
| --- | --- | --- | --- | --- | --- | --- | --- | --- | --- | --- | --- | --- | --- | --- |
| CTO |  |  |  |  |  |  |  |  |  |  |  |  |  |  |
| Time |  | 12821.16 |  | 1,74 |  | 12821.158 |  | 62.046 |  | **< .001** |  | 0.059 |  | < 0.001 |
| Group |  | 458.53 |  | 1,74 |  | 458.526 |  | 0.178 |  | .674 |  | 0.002 |  | 2.368 |
| Time x Group |  | 9.50 |  | 1,74 |  | 9.500 |  | 0.046 |  | .831 |  | < 0.001 |  | 3.201 |
| E% |  |  |  |  |  |  |  |  |  |  |  |  |  |  |
| Time |  | 494.19 |  | 1,74 |  | 494.193 |  | 16.770 |  | **< .001** |  | 0.035 |  | 0.007 |
| Group |  | 0.04 |  | 1,74 |  | 0.043 |  | < 0.001 |  | .987 |  | < 0.001 |  | 3.318 |
| Time x Group |  | 15.93 |  | 1,74 |  | 15.925 |  | 0.540 |  | .465 |  | 0.001 |  | 3.441 |
| CP |  |  |  |  |  |  |  |  |  |  |  |  |  |  |
| Time |  | 23800.03 |  | 1,74 |  | 23800.026 |  | 152.060 |  | **< .001** |  | 0.108 |  | < .001 |
| Group |  | 164.24 |  | 1,74 |  | 164.237 |  | 0.066 |  | .798 |  | < .001 |  | 2.029 |
| Time x Group |  | 172.66 |  | 1,74 |  | 172.658 |  | 1.103 |  | .297 |  | < .001 |  | 1.783 |
| *Note.* Type III Sum of Squares (SS); Sample sizes were *n* = 38 for the open-label-placebo group and *n* = 38 for the control group; BF_01_ = evidence for the null vs. the alternative hypothesis; CTO = number of finished target objects; E% = error percent; CP = concentration performance. The alpha error probability corrected for multiple comparisons in 14 objective tests was *p* = .05/14 = .004. | | | | | | | | | | | | | | |

## Subjective task outcomes

Tables S8-10 show the analyses of the subjective visual analogue scales. Regarding satisfaction, we additionally observed a significant main effect of time (albeit only anecdotal evidence for such an effect according to the Bayesian analyses) in the d2 task and the 2-back task (all *p*’s < .024). All other effects/tasks were not significant (all *p*'s > .230) and Bayesian analyses supported the null over the alternative hypothesis with substantial evidence.

*Table S8. Mixed ANOVAs and BF_01_’s for subjective effort in the five objective tasks.*

| Measure & Effect | | $n$(OLP/CTR) | $SS$ | | *df*‘s | | Mean Square | | $F$ | | $p$ | | $ƞ^{2}$ | | BF_01_ |
| --- | --- | --- | --- | --- | --- | --- | --- | --- | --- | --- | --- | --- | --- | --- | --- |
| Stroop task |  | 38/39 |  |  |  |  |  |  |  |  |  |  |  |  |  |
| Time |  |  | 622.46 |  | 1,75 |  | 622.459 |  | 1.862 |  | .176 |  | 0.008 |  | 3.4622 |
| Group |  |  | 26.62 |  | 1,75 |  | 26.623 |  | 0.037 |  | .848 |  | < 0.001 |  | 5.566 |
| Time x Group |  |  | 215.00 |  | 1,75 |  | 215.004 |  | 0.643 |  | .425 |  | 0.003 |  | 13.012 |
| 2-back task |  | 38/37 |  |  |  |  |  |  |  |  |  |  |  |  |  |
| Time |  |  | 4657.16 |  | 1,73 |  | 4657.163 |  | 26.875 |  | **< .001** |  | 0.059 |  | 0.001 |
| Group |  |  | 241.05 |  | 1,73 |  | 241.047 |  | 0.286 |  | .594 |  | 0.003 |  | 2.935 |
| Time x Group |  |  | 132.79 |  | 1,73 |  | 132.789 |  | 0.766 |  | .384 |  | 0.002 |  | 2.839 |
| Dual task |  | 38/39 |  |  |  |  |  |  |  |  |  |  |  |  |  |
| Time |  |  | 1247.87 |  | 1,75 |  | 1247.872 |  | 3.920 |  | .051 |  | 0.016 |  | 1.285 |
| Group |  |  | 1092.99 |  | 1,75 |  | 1092.988 |  | 1.577 |  | .213 |  | 0.014 |  | 2.802 |
| Time x Group |  |  | 13.33 |  | 1,75 |  | 13.327 |  | 0.042 |  | .838 |  | 0.001 |  | 6.168 |
| Learning task |  | 37/36 |  |  |  |  |  |  |  |  |  |  |  |  |  |
| Time |  |  | 2476.587 |  | 1,71 |  | 2476.587 |  | 5.323 |  | .024 |  | 0.030 |  | 0.575 |
| Group |  |  | 495.666 |  | 1,71 |  | 495.666 |  | 0.749 |  | .390 |  | 0.006 |  | 4.231 |
| Time x Group |  |  | 251.820 |  | 1,71 |  | 251.820 |  | 0.541 |  | .464 |  | 0.003 |  | 5.135 |
| d2 task |  | 36/39 |  |  |  |  |  |  |  |  |  |  |  |  |  |
| Time |  |  | 7394.35 |  | 1,73 |  | 7394.347 |  | 27.647 |  | **< .001** |  | 0.083 |  | 0.001 |
| Group |  |  | 1.85 |  | 1,73 |  | 1.849 |  | 0.002 |  | .963 |  | < 0.001 |  | 3.975 |
| Time x Group |  |  | 66.77 |  | 1,73 |  | 66.774 |  | 0.250 |  | .619 |  | 0.007 |  | 4.280 |
| *Note.* Type III Sum of Squares (SS); BF_01_ = evidence for the null vs. the alternative hypothesis. The alpha error probability corrected for multiple comparisons in 15 subjective tests was *p* = .05/15 = .003. | | | | | | | | | | | | | | | |

Table S9. Mixed ANOVAs and BF_01_’s for subjective satisfaction in the five objective tasks.

| Measure & Effect | | $n$(OLP/CTR) | $SS$ | | *df*‘s | | Mean Square | | $F$ | | $p$ | | $ƞ^{2}$ | | BF_01_ |
| --- | --- | --- | --- | --- | --- | --- | --- | --- | --- | --- | --- | --- | --- | --- | --- |
| Stroop task |  | 38/39 |  |  |  |  |  |  |  |  |  |  |  |  |  |
| Time |  |  | 24.63 |  | 1,75 |  | 24.632 |  | 0.051 |  | .821 |  | 0.003 |  | 7.845 |
| Group |  |  | 746.99 |  | 1,75 |  | 746.995 |  | 1.465 |  | .230 |  | 0.010 |  | 3.680 |
| Time x Group |  |  | 6.32 |  | 1,75 |  | 6.320 |  | 0.013 |  | .909 |  | < 0.001 |  | 29.193 |
| 2-back task |  | 38/37 |  |  |  |  |  |  |  |  |  |  |  |  |  |
| Time |  |  | 3756.00 |  | 1,73 |  | 3756.003 |  | 9.884 |  | **.002** |  | 0.050 |  | 0.040 |
| Group |  |  | 260.00 |  | 1,73 |  | 260.000 |  | 0.450 |  | .505 |  | 0.003 |  | 5.077 |
| Time x Group |  |  | 816.56 |  | 1,73 |  | 816.563 |  | 2.149 |  | .147 |  | 0.011 |  | 4.457 |
| Dual task |  | 38/39 |  |  |  |  |  |  |  |  |  |  |  |  |  |
| Time |  |  | 664.94 |  | 1,75 |  | 664.939 |  | 2.867 |  | .095 |  | 0.009 |  | 0.593 |
| Group |  |  | 751.35 |  | 1,75 |  | 751.350 |  | 1.010 |  | .318 |  | 0.010 |  | 0.429 |
| Time x Group |  |  | 951.59 |  | 1,75 |  | 951.589 |  | 4.103 |  | .046 |  | 0.013 |  | 0.628 |
| Learning task |  | 37/36 |  |  |  |  |  |  |  |  |  |  |  |  |  |
| Time |  |  | 191.72 |  | 1,71 |  | 191.715 |  | 0.647 |  | .424 |  | 0.003 |  | 0.231 |
| Group |  |  | 1201.52 |  | 1,71 |  | 1201.519 |  | 1.778 |  | .187 |  | 0.017 |  | 0.448 |
| Time x Group |  |  | 1177.74 |  | 1,71 |  | 1177.743 |  | 3.974 |  | **.050** |  | 0.016 |  | 0.358 |
| d2 |  | 36/39 |  |  |  |  |  |  |  |  |  |  |  |  |  |
| Time |  |  | 1286.63 |  | 1,73 |  | 1286.631 |  | 4.073 |  | **.047** |  | 0.022 |  | 0.637 |
| Group |  |  | 1860.97 |  | 1,73 |  | 1860.974 |  | 4.412 |  | **.039** |  | 0.032 |  | 0.742 |
| Time x Group |  |  | 978.47 |  | 1,73 |  | 978.471 |  | 3.097 |  | .083 |  | 0.017 |  | 0.686 |
| *Note.* Type III Sum of Squares (SS); BF_01_ = evidence for the null vs. the alternative hypothesis. The alpha error probability corrected for multiple comparisons in 15 subjective tests was *p* = .05/15 = .003. | | | | | | | | | | | | | | | |

Table S10. Welch’s t-tests and BF_01_’s for subjective change in the five objective tasks.

| Measure | | $n$(OLP/CTR) | $t$ | *df‘s* | | $p$ | | $ƞ^{2}$ |  | | BF_01_ |
| --- | --- | --- | --- | --- | --- | --- | --- | --- | --- | --- | --- |
| Stroop task |  | 38/39 | 0.02 | 73.526 |  | .981 |  | < 0.001 |  |  | 4.232 |
| 2-back task |  | 38/38 | -1.63 | 69.140 |  | .108 |  | 0.034 |  |  | 1.351 |
| Dual task |  | 38/39 | -1.23 | 74.477 |  | .224 |  | 0.019 |  |  | 2.209 |
| Learning task |  | 37/38 | 0.67 | 72.607 |  | .503 |  | 0.005 |  |  | 3.445 |
| d2 task |  | 36/39 | 0.20 | 71.293 |  | .843 |  | < 0.001 |  |  | 4.112 |
| *Note.* BF_01_ = evidence for the null vs. the alternative hypothesis. The alpha error probability corrected for multiple comparisons in 15 subjective tests was *p* = .05/15 = .003. | | | | | | | | | | | |

## Subjective state questionnaires

Table S11 shows the analyses of the subjective questionnaires. We observed a significant main effect of time for motivation and substantial evidence for this effect (*p* = .001; all other effects n.s.), whereby motivation decreased similarly across both groups from baseline to test. We also found a significant main effect of time for fatigue and anecdotal evidence for this effect (*p* = .033; all other effects n.s.), whereby fatigue increased similarly in both groups from baseline to test. Furthermore, we found a significant main effect of time for joy and anecdotal evidence for this effect (*p* = .029; all other effects n.s.), whereby joy decreased similarly in both groups from baseline to test. In the remaining subjective parameters, we neither found main effects of group and time, nor interactions between these two factors: anxiety/depression facets (emotionality, worry, anhedonia, and dysthymia; all *p*'s > .303); sleep quality (all *p*'s > .314); activity (all *p*'s > .070) and sports indices (all *p*'s > .316); mood states (depression, anger, confusion, and total score; all *p*'s > .065); and stress facets (worries, tension, demands, and total score; all *p*'s > .376). These results were again supported by our Bayesian analyses showing varying but mostly substantial evidence for the null compared to the alternative hypothesis.

Table S11. Mixed ANOVAs and BF_01_’s for subjective questionnaires.

| Measure & Effect | | $n$  (OLP/CTR) | $SS$ | | *df*‘s | | Mean Square | | $F$ | | $p$ | | $ƞ^{2}$ | | BF_01_ |
| --- | --- | --- | --- | --- | --- | --- | --- | --- | --- | --- | --- | --- | --- | --- | --- |
| STADI (state) Emotionality |  | 39/39 |  |  |  |  |  |  |  |  |  |  |  |  |  |
| Time |  |  | 0.04 |  | 1,76 |  | 0.037 |  | 1.077 |  | .303 |  | 0.004 |  | 4.850 |
| Group |  |  | 0.001 |  | 1,76 |  | 0.001 |  | 0.012 |  | .915 |  | 0.001 |  | 5.321 |
| Time x Group |  |  | 0.04 |  | 1,76 |  | 0.037 |  | 1.077 |  | .303 |  | 0.004 |  | 13.584 |
| STADI (state) Worry |  | 39/39 |  |  |  |  |  |  |  |  |  |  |  |  |  |
| Time |  |  | 0.02 |  | 1,76 |  | 0.016 |  | 0.358 |  | .552 |  | 0.001 |  | 7.191 |
| Group |  |  | 0.04 |  | 1,76 |  | 0.037 |  | 0.361 |  | .550 |  | 0.003 |  | 4.842 |
| Time x Group |  |  | 0.02 |  | 1,76 |  | 0.016 |  | 0.358 |  | .552 |  | 0.001 |  | 24.589 |
| STADI (state) Anhedonia |  | 39/39 |  |  |  |  |  |  |  |  |  |  |  |  |  |
| Time |  |  | 0.002 |  | 1,76 |  | 0.002 |  | 0.006 |  | .937 |  | < 0.001 |  | 8.209 |
| Group |  |  | 0.04 |  | 1,76 |  | 0.043 |  | 0.645 |  | .424 |  | 0.005 |  | 4.940 |
| Time x Group |  |  | 0.01 |  | 1,76 |  | 0.013 |  | 0.309 |  | .580 |  | 0.002 |  | 28.492 |
| STADI (state) Dysthymia |  | 39/39 |  |  |  |  |  |  |  |  |  |  |  |  |  |
| Time |  |  | 0.002 |  | 1,76 |  | 0.002 |  | 0.121 |  | .729 |  | 0.005 |  | 7.331 |
| Group |  |  | 0.06 |  | 1,76 |  | 0.058 |  | 1.851 |  | .178 |  | 0.015 |  | 3.013 |
| Time x Group |  |  | 0.01 |  | 1,76 |  | 0.006 |  | 0.336 |  | .564 |  | 0.002 |  | 19.487 |
| PSQI |  |  |  |  |  |  |  |  |  |  |  |  |  |  |  |
| Time |  |  | 0.64 |  | 1,76 |  | 0.641 |  | 0.257 |  | .614 |  | 0.005 |  | 7.102 |
| Group |  |  | 3.10 |  | 1,76 |  | 3.103 |  | 0.225 |  | .637 |  | 0.002 |  | 3.808 |
| Time x Group |  |  | 2.56 |  | 1,76 |  | 2.564 |  | 1.027 |  | .314 |  | 0.002 |  | 15.492 |
| BSA Activity |  | 39/39 |  |  |  |  |  |  |  |  |  |  |  |  |  |
| Time |  |  | 9462.98 |  | 1,76 |  | 9462.981 |  | 0.082 |  | .776 |  | 0.001 |  | 6.651 |
| Group |  |  | 85634.78 |  | 1,76 |  | 85634.776 |  | 0.101 |  | .751 |  | 0.001 |  | 3.156 |
| Time x Group |  |  | 404634.78 |  | 1,76 |  | 404634.776 |  | 3.498 |  | .065 |  | 0.005 |  | 5.712 |
| BSA Sport |  | 39/39 |  |  |  |  |  |  |  |  |  |  |  |  |  |
| Time |  |  | 11568.05 |  | 1,76 |  | 11568.050 |  | 1.033 |  | .316 |  | 0.005 |  | 3.726 |
| Group |  |  | 34528.05 |  | 1,76 |  | 34528.050 |  | 0.789 |  | .380 |  | 0.016 |  | 2.615 |
| Time x Group |  |  | 156.80 |  | 1,76 |  | 156.800 |  | 0.014 |  | .906 |  | < 0.001 |  | 8.152 |
| POMS Total |  | 39/39 |  |  |  |  |  |  |  |  |  |  |  |  |  |
| Time |  |  | 253.85 |  | 1,76 |  | 253.853 |  | 1.470 |  | .229 |  | 0.004 |  | 4.014 |
| Group |  |  | 668.78 |  | 1,76 |  | 668.776 |  | 1.006 |  | .319 |  | 0.010 |  | 3.146 |
| Time x Group |  |  | 55.44 |  | 1,76 |  | 55.442 |  | 0.321 |  | .573 |  | 0.008 |  | 12.298 |
| POMS Depression |  | 39/39 |  |  |  |  |  |  |  |  |  |  |  |  |  |
| Time |  |  | 3.39 |  | 1,76 |  | 3.391 |  | 0.192 |  | .662 |  | 0.005 |  | 7.388 |
| Group |  |  | 4.67 |  | 1,76 |  | 4.673 |  | 0.068 |  | .795 |  | 0.007 |  | 4.512 |
| Time x Group |  |  | 15.39 |  | 1,76 |  | 15.391 |  | 0.872 |  | .353 |  | 0.002 |  | 20.393 |
| POMS Anger |  | 39/39 |  |  |  |  |  |  |  |  |  |  |  |  |  |
| Time |  |  | 0.92 |  | 1,76 |  | 0.923 |  | 0.131 |  | .719 |  | 0.004 |  | 7.832 |
| Group |  |  | 5.03 |  | 1,76 |  | 5.026 |  | 0.230 |  | .633 |  | 0.002 |  | 4.725 |
| Time x Group |  |  | 0.10 |  | 1,76 |  | 0.103 |  | 0.015 |  | .904 |  | < 0.001 |  | 29.168 |
| POMS Motivation |  | 39/39 |  |  |  |  |  |  |  |  |  |  |  |  |  |
| Time |  |  | 176.64 |  | 1,76 |  | 176.641 |  | 11.677 |  | .001 |  | 0.027 |  | 0.047 |
| Group |  |  | 72.03 |  | 1,76 |  | 72.026 |  | 1.085 |  | .301 |  | 0.011 |  | 2.468 |
| Time x Group |  |  | 1.64 |  | 1,76 |  | 1.641 |  | 0.108 |  | .743 |  | 0.002 |  | 3.572 |
| POMS Fatigue |  | 39/39 |  |  |  |  |  |  |  |  |  |  |  |  |  |
| Time |  |  | 46.31 |  | 1,76 |  | 46.314 |  | 4.732 |  | .033 |  | 0.011 |  | 0.953 |
| Group |  |  | 73.39 |  | 1,76 |  | 73.391 |  | 1.709 |  | .195 |  | 0.018 |  | 2.162 |
| Time x Group |  |  | 0.31 |  | 1,76 |  | 0.314 |  | 0.032 |  | .858 |  | < 0.001 |  | 4.511 |
| POMS Confusion |  | 39/39 |  |  |  |  |  |  |  |  |  |  |  |  |  |
| Time |  |  | 14.77 |  | 1,76 |  | 14.769 |  | 3.511 |  | .065 |  | 0.008 |  | 1.621 |
| Group |  |  | 16.03 |  | 1,76 |  | 16.026 |  | 0.861 |  | .357 |  | 0.009 |  | 2.754 |
| Time x Group |  |  | 6.56 |  | 1,76 |  | 6.564 |  | 1.561 |  | .215 |  | 0.004 |  | 3.625 |
| PSQ Worries |  | 39/39 |  |  |  |  |  |  |  |  |  |  |  |  |  |
| Time |  |  | 72.95 |  | 1,76 |  | 72.953 |  | 0.499 |  | .482 |  | 0.007 |  | 6.498 |
| Group |  |  | 411.42 |  | 1,76 |  | 411.418 |  | 0.363 |  | .549 |  | 0.004 |  | 3.125 |
| Time x Group |  |  | 10.27 |  | 1,76 |  | 10.267 |  | 0.070 |  | .792 |  | 0.001 |  | 18.599 |
| PSQ Joy |  | 39/39 |  |  |  |  |  |  |  |  |  |  |  |  |  |
| Time |  |  | 629.53 |  | 1,76 |  | 629.532 |  | 4.949 |  | .029 |  | 0.010 |  | 0.875 |
| Group |  |  | 0.281 |  | 1,76 |  | 0.281 |  | 0.003 |  | .984 |  | < 0.001 |  | 3.536 |
| Time x Group |  |  | 82.375 |  | 1,76 |  | 82.375 |  | 0.648 |  | .423 |  | 0.001 |  | 5.070 |
| PSQ Demands |  | 39/39 |  |  |  |  |  |  |  |  |  |  |  |  |  |
| Time |  |  | < 0.001 |  | 1,76 |  | < 0.001 |  | < 0.001 |  | > .999 |  | < 0.001 |  | 8.099 |
| Group |  |  | 137.79 |  | 1,76 |  | 137.785 |  | 0.152 |  | .697 |  | 0.002 |  | 3.990 |
| Time x Group |  |  | 113.92 |  | 1,76 |  | 113.920 |  | 0.602 |  | 0.440 |  | 0.001 |  | 19.710 |
| PSQ Total |  | 39/39 |  |  |  |  |  |  |  |  |  |  |  |  |  |
| Time |  |  | 51.90 |  | 1,76 |  | 51.900 |  | 0.793 |  | 0.376 |  | 0.009 |  | 5.676 |
| Group |  |  | 87.30 |  | 1,76 |  | 87.301 |  | 0.132 |  | 0.717 |  | 0.002 |  | 3.222 |
| Time x Group |  |  | 0.64 |  | 1,76 |  | 0.644 |  | 0.010 |  | 0.921 |  | < 0.001 |  | 17.804 |
| *Note.* Type III Sum of Squares (SS); BF_01_ = evidence for the null vs. the alternative hypothesis. STADI = State-Trait-Anxiety-Depression-Inventory; PSQI = Pittsburg Sleep Quality Index; BSA = Bewegungs- und Sportaktivitätsfragebogen [Movement and Sports Activity Questionnaire]; POMS = Profile of Mood States; PSQ = Perceived Stress Questionnaire. | | | | | | | | | | | | | | | |

## Gender effects

We explored gender as an additional factor in our primary analyses of objective and subjective measures. In contrast to the analyses without gender, we found the following additional uncorrected effects:

- Main effect of gender in the d2 task (*p* = .021; finished target objects: F > M)
- Time x gender interaction in the d2 task (*p* = .048; concentration performance: F increased more than M from baseline to test)
- Time x group x gender interaction in the subjective satisfaction in the d2 task (*p* = .031; OLP and CTR groups differ in satisfaction at baseline only for females)
- Time x gender interaction in the subjective effort in the 2-back task (*p* = .008; F perceive task to be more effortful than M at baseline and show a stronger decrease in effort than M from baseline to test)

Please note that none of these results survived correcting for multiple comparison and none of the previously reported results changed. In sum, no effect of gender on group differences or interactions including the factor group were observed.

Table S12. Mixed ANOVAs and BF_01_’s for the three outcomes of the Stroop task, including gender.

| Measure & Effect |  | $SS$ |  | *df*‘s |  | Mean Square |  | $F$ |  | $p$ |  | $ƞ^{2}$ |  |  |
| --- | --- | --- | --- | --- | --- | --- | --- | --- | --- | --- | --- | --- | --- | --- |
| Reaction time |  |  |  |  |  |  |  |  |  |  |  |  |  |  |
| Time |  | 17791.42 |  | 1,72 |  | 17791.420 |  | 1.951 |  | .167 |  | 0.001 |  |  |
| Group |  | 66995.94 |  | 1,72 |  | 66995.939 |  | 0.454 |  | .502 |  | 0.005 |  |  |
| Congruency |  | 970802.50 |  | 1,72 |  | 970802.498 |  | 205.920 |  | **< .001** |  | 0.075 |  |  |
| Gender |  | 27000.97 |  | 1,72 |  | 27000.967 |  | 0.183 |  | .670 |  | 0.002 |  |  |
| Time x Group |  | 241.31 |  | 1,72 |  | 241.310 |  | 0.026 |  | .871 |  | < 0.001 |  |  |
| Time x Gender |  | 7346.14 |  | 1,72 |  | 7346.137 |  | 0.806 |  | .372 |  | 0.005 |  |  |
| Group x Gender |  | 28285.99 |  | 1,72 |  | 28285.990 |  | 0.192 |  | .663 |  | 0.002 |  |  |
| Congruency x Group |  | 2651.46 |  | 1,72 |  | 2651.455 |  | 0.562 |  | .456 |  | 0.002 |  |  |
| Congruency x Gender |  | 785.77 |  | 1,72 |  | 785.769 |  | 0.167 |  | .684 |  | < 0.001 |  |  |
| Time x Congruency |  | 907.68 |  | 1,72 |  | 907.678 |  | 0.477 |  | .492 |  | < 0.001 |  |  |
| Time x Group x Gender |  | 13062.56 |  | 1,72 |  | 13062.563 |  | 1.432 |  | .235 |  | 0.001 |  |  |
| Co x Group x Gender |  | 4623.44 |  | 1,72 |  | 4623.440 |  | 0.981 |  | .325 |  | 0.003 |  |  |
| Time x Co x Group |  | 1371.24 |  | 1,72 |  | 1371.243 |  | 0.721 |  | .399 |  | 0.001 |  |  |
| Time x Co x Gender |  | 1944.51 |  | 1,72 |  | 1944.514 |  | 1.022 |  | .315 |  | 0.001 |  |  |
| Time x Co x Gr x Ge |  | 1.02 |  | 1,72 |  | 1.023 |  | 5.381e-4 |  | .982 |  | < 0.001 |  |  |
| Accuracy |  |  |  |  |  |  |  |  |  |  |  |  |  |  |
| Time |  | 0.005 |  | 1,73 |  | 0.005 |  | 0.749 |  | .390 |  | 0.002 |  |  |
| Group |  | < 0.001 |  | 1,73 |  | < 0.001 |  | 0.001 |  | .972 |  | < 0.001 |  |  |
| Congruency |  | 0.151 |  | 1,73 |  | 0.151 |  | 16.422 |  | **< .001** |  | 0.056 |  |  |
| Gender |  | 0.004 |  | 1,73 |  | 0.004 |  | 0.310 |  | .579 |  | 0.001 |  |  |
| Time x Group |  | 0.010 |  | 1,73 |  | 0.010 |  | 1.668 |  | .201 |  | 0.004 |  |  |
| Time x Gender |  | 0.008 |  | 1,73 |  | 0.008 |  | 0.136 |  | .714 |  | 0.003 |  |  |
| Group x Gender |  | < 0.001 |  | 1,73 |  | < 0.001 |  | 0.003 |  | .954 |  | < 0.001 |  |  |
| Congruency x Group |  | 0.007 |  | 1,73 |  | 0.006 |  | 0.074 |  | .786 |  | 0.002 |  |  |
| Congruency x Gender |  | 0.005 |  | 1,73 |  | 0.005 |  | 0.547 |  | .462 |  | 0.002 |  |  |
| Time x Congruency |  | 0.007 |  | 1,73 |  | 0.007 |  | 1.110 |  | .296 |  | 0.003 |  |  |
| Time x Group x Gender |  | 0.022 |  | 1,73 |  | 0.022 |  | 3.681 |  | .059 |  | 0.008 |  |  |
| Co x Group x Gender |  | < 0.001 |  | 1,73 |  | < 0.001 |  | 0.007 |  | .932 |  | < 0.001 |  |  |
| Time x Co x Group |  | 0.009 |  | 1,73 |  | 0.009 |  | 1.458 |  | .231 |  | 0.003 |  |  |
| Time x Co x Gender |  | < 0.001 |  | 1,73 |  | < 0.001 |  | 0.011 |  | .917 |  | < 0.001 |  |  |
| Time x Co x Gr x Ge |  | 0.017 |  | 1,73 |  | 0.017 |  | 2.768 |  | .100 |  | 0.006 |  |  |
| Stroop effect |  |  |  |  |  |  |  |  |  |  |  |  |  |  |
| Time |  | 1815.36 |  | 1,72 |  | 1815.355 |  | 0.477 |  | .492 |  | 0.002 |  |  |
| Group |  | 5302.91 |  | 1,72 |  | 5302.910 |  | 0.562 |  | .456 |  | 0.005 |  |  |
| Gender |  | 1571.54 |  | 1,72 |  | 1571.538 |  | 0.167 |  | .684 |  | 0.002 |  |  |
| Time x Group |  | 2742.49 |  | 1,72 |  | 2742.487 |  | 0.721 |  | .399 |  | 0.003 |  |  |
| Time x Gender |  | 3889.03 |  | 1,72 |  | 3889.028 |  | 1.022 |  | .315 |  | 0.004 |  |  |
| Group x Ge |  | 9246.88 |  | 1,72 |  | 9246.880 |  | 0.981 |  | .325 |  | 0.009 |  |  |
| T x Gr x Ge |  | 2.05 |  | 1,72 |  | 2.047 |  | 0.005 |  | .982 |  | < 0.001 |  |  |
| *Note.* Type III Sum of Squares (SS); Sample sizes were *n* = 38 for the open-label-placebo group and *n* = 38/39 for the control group; BF_01_ = evidence for the null vs. the alternative hypothesis; C = congruency; T = time; G = group. | | | | | | | | | | | | | | |

Table S13. Mixed ANOVAs and BF_01_’s for the three outcomes of the dual task, including gender.

| Measure & Effect |  | $SS$ |  | *df*‘s |  | Mean Square |  | $F$ |  | $p$ |  | $ƞ^{2}$ |  |  |
| --- | --- | --- | --- | --- | --- | --- | --- | --- | --- | --- | --- | --- | --- | --- |
| Reaction time |  |  |  |  |  |  |  |  |  |  |  |  |  |  |
| Time |  | 32542.08 |  | 1,72 |  | 32542.081 |  | 12.577 |  | **< .001** |  | 0.010 |  |  |
| Group |  | 404.77 |  | 1,72 |  | 404.766 |  | 0.028 |  | .867 |  | 0.001 |  |  |
| Target |  | 1.463e+6 |  | 1,72 |  | 1.463e+6 |  | 313.198 |  | **< .001** |  | 0.451 |  |  |
| Gender |  | 4093.94 |  | 1,72 |  | 4093.943 |  | 0.285 |  | .595 |  | 0.001 |  |  |
| Time x Group |  | 1475.93 |  | 1,72 |  | 1475.926 |  | 0.570 |  | .453 |  | 0.004 |  |  |
| Time x Gender |  | 49.67 |  | 1,72 |  | 49.672 |  | 0.019 |  | .890 |  | < 0.001 |  |  |
| Group x Gender |  | 12222.80 |  | 1,72 |  | 12222.801 |  | 0.850 |  | .360 |  | 0.004 |  |  |
| Target x Group |  | 472.61 |  | 1,72 |  | 472.612 |  | 0.101 |  | .751 |  | 0.001 |  |  |
| Target x Gender |  | 4545.62 |  | 1,72 |  | 4545.622 |  | 0.973 |  | .327 |  | 0.001 |  |  |
| Time x Target |  | 585.65 |  | 1,72 |  | 585.652 |  | 0.258 |  | .613 |  | 0.001 |  |  |
| Time x Group x Gender |  | 1584.94 |  | 1,72 |  | 1584.939 |  | 0.613 |  | .436 |  | 0.004 |  |  |
| Target x Group x Gender |  | 54.22 |  | 1,72 |  | 54.223 |  | 0.012 |  | .914 |  | < 0.001 |  |  |
| Time x Target x Group |  | 8.13 |  | 1,72 |  | 8.130 |  | 0.004 |  | .952 |  | < 0.001 |  |  |
| Time x Target x Gender |  | 1.04 |  | 1,72 |  | 1.043 |  | 0.004 |  | .983 |  | < 0.001 |  |  |
| Time x Target x Gr x Ge |  | 619.46 |  | 1,72 |  | 619.460 |  | 0.273 |  | .603 |  | 0.001 |  |  |
| Hits |  |  |  |  |  |  |  |  |  |  |  |  |  |  |
| Time |  | 12.01 |  | 1,73 |  | 12.012 |  | 2.173 |  | .145 |  | 0.009 |  |  |
| Group |  | 3.21 |  | 1,73 |  | 3.207 |  | 0.363 |  | .548 |  | 0.002 |  |  |
| Target |  | 26.55 |  | 1,73 |  | 26.545 |  | 12.959 |  | **< .001** |  | 0.020 |  |  |
| Gender |  | 3.46 |  | 1,73 |  | 3.455 |  | 0.392 |  | .533 |  | 0.003 |  |  |
| Time x Group |  | 7.12 |  | 1,73 |  | 7.122 |  | 1.289 |  | .260 |  | 0.005 |  |  |
| Time x Gender |  | 10.56 |  | 1,73 |  | 10.558 |  | 1.910 |  | .171 |  | 0.008 |  |  |
| Group x Gender |  | 2.33 |  | 1,73 |  | 2.330 |  | 0.264 |  | .609 |  | 0.002 |  |  |
| Target x Group |  | 0.13 |  | 1,73 |  | 0.132 |  | 0.065 |  | .800 |  | 0.001 |  |  |
| Target x Gender |  | 0.10 |  | 1,73 |  | 0.100 |  | 0.049 |  | .825 |  | < 0.001 |  |  |
| Time x Target |  | 0.09 |  | 1,73 |  | 0.093 |  | 0.134 |  | .715 |  | < 0.001 |  |  |
| Time x Group x Gender |  | 4.68 |  | 1,73 |  | 4.681 |  | 0.847 |  | .360 |  | 0.004 |  |  |
| Target x Group x Gender |  | 1.68 |  | 1,73 |  | 1.680 |  | 0.820 |  | .368 |  | 0.001 |  |  |
| Time x Target x Group |  | 1.40 |  | 1,73 |  | 1.397 |  | 2.008 |  | .161 |  | 0.001 |  |  |
| Time x Target x Gender |  | 1.84 |  | 1,73 |  | 1.835 |  | 2.638 |  | .109 |  | 0.001 |  |  |
| Time x Target x Gr x Ge |  | 0.06 |  | 1,73 |  | 0.059 |  | 0.085 |  | .771 |  | < 0.001 |  |  |
| Misses |  |  |  |  |  |  |  |  |  |  |  |  |  |  |
| Time |  | 11.91 |  | 1,73 |  | 11.905 |  | 2.233 |  | .139 |  | 0.010 |  |  |
| Group |  | 4.10 |  | 1,73 |  | 4.097 |  | 0.486 |  | .488 |  | 0.003 |  |  |
| Target |  | 11.49 |  | 1,73 |  | 11.490 |  | 6.626 |  | **.012** |  | 0.009 |  |  |
| Gender |  | 2.45 |  | 1,73 |  | 2.446 |  | 0.290 |  | .592 |  | 0.002 |  |  |
| Time x Group |  | 8.66 |  | 1,73 |  | 8.655 |  | 1.623 |  | .207 |  | 0.007 |  |  |
| Time x Gender |  | 10.00 |  | 1,73 |  | 9.996 |  | 1.875 |  | .175 |  | 0.008 |  |  |
| Group x Gender |  | 2.23 |  | 1,73 |  | 2.234 |  | 0.265 |  | .608 |  | 0.002 |  |  |
| Target x Group |  | 0.36 |  | 1,73 |  | 0.356 |  | 0.205 |  | .652 |  | 0.002 |  |  |
| Target x Gender |  | 0.004 |  | 1,73 |  | 0.004 |  | 0.002 |  | .987 |  | < 0.001 |  |  |
| Time x Target |  | 0.10 |  | 1,73 |  | 0.103 |  | 0.152 |  | .698 |  | < 0.001 |  |  |
| Time x Group x Gender |  | 3.19 |  | 1,73 |  | 3.194 |  | 0.599 |  | .441 |  | 0.003 |  |  |
| Target x Group x Gender |  | 1.76 |  | 1,73 |  | 1.763 |  | 1.016 |  | .317 |  | 0.001 |  |  |
| Time x Target x Group |  | 2.12 |  | 1,73 |  | 2.117 |  | 3.122 |  | .081 |  | 0.002 |  |  |
| Time x Target x Gender |  | 1.61 |  | 1,73 |  | 1.605 |  | 2.367 |  | .128 |  | 0.001 |  |  |
| Time x Target x Gr x Ge |  | 0.02 |  | 1,73 |  | 0.018 |  | 0.026 |  | .872 |  | < 0.001 |  |  |
| *Note.* Type III Sum of Squares (SS); Sample sizes were *n* = 37/38 for the open-label-placebo group and *n* = 39 for the control group; BF_01_ = evidence for the null vs. the alternative hypothesis; Ta = Target; Ti = time; G = group. | | | | | | | | | | | | | | |

*Table S14. Mixed ANOVAs and BF_01_’s for the three outcomes of the 2-back task, including gender.*

| Measure & Effect | | $SS$ | | *df*‘s | | Mean Square | | $F$ | | $p$ | | $ƞ^{2}$ | |
| --- | --- | --- | --- | --- | --- | --- | --- | --- | --- | --- | --- | --- | --- |
| Reaction time |  |  |  |  |  |  |  |  |  |  |  |  |  |
| Time |  | 1108.33 |  | 1,71 |  | 1108.332 |  | 0.137 |  | .712 |  | 0.005 |  |
| Group |  | 729.12 |  | 1,71 |  | 729.122 |  | 0.033 |  | .856 |  | 0.003 |  |
| Gender |  | 657.27 |  | 1,71 |  | 657.274 |  | 0.030 |  | .863 |  | 0.003 |  |
| Time x Group |  | 390.57 |  | 1,71 |  | 390.566 |  | 0.048 |  | .826 |  | 0.001 |  |
| Time x Gender |  | 88.83 |  | 1,71 |  | 88.828 |  | 0.011 |  | .917 |  | < 0.001 |  |
| Group x Ge |  | 48753.47 |  | 1,71 |  | 48753.474 |  | 2.228 |  | .140 |  | 0.022 |  |
| T x Gr x Ge |  | 5185.00 |  | 1,71 |  | 5185.000 |  | 0.643 |  | .425 |  | 0.002 |  |
| Hit rate |  |  |  |  |  |  |  |  |  |  |  |  |  |
| Time |  | 0.03 |  | 1,72 |  | 0.027 |  | 3.444 |  | .068 |  | 0.006 |  |
| Group |  | 0.01 |  | 1,72 |  | 0.005 |  | 0.010 |  | .920 |  | 0.001 |  |
| Gender |  | 0.02 |  | 1,72 |  | 0.019 |  | 0.347 |  | .557 |  | 0.004 |  |
| Time x Group |  | 0.003 |  | 1,72 |  | 0.003 |  | 0.385 |  | .537 |  | 0.006 |  |
| Time x Gender |  | 0.02 |  | 1,72 |  | 0.019 |  | 2.450 |  | .122 |  | 0.004 |  |
| Group x Ge |  | 0.02 |  | 1,72 |  | 0.018 |  | 0.324 |  | .571 |  | 0.004 |  |
| T x Gr x Ge |  | 0.01 |  | 1,72 |  | 0.011 |  | 1.387 |  | .243 |  | 0.002 |  |
| d-prime |  |  |  |  |  |  |  |  |  |  |  |  |  |
| Time |  | 1.11 |  | 1,72 |  | 1.114 |  | 5.051 |  | **.028** |  | 0.010 |  |
| Group |  | 0.07 |  | 1,72 |  | 0.067 |  | 0.053 |  | .818 |  | 0.006 |  |
| Gender |  | 0.01 |  | 1,72 |  | 0.008 |  | 0.007 |  | .935 |  | < 0.001 |  |
| Time x Group |  | 0.12 |  | 1,72 |  | 0.115 |  | 0.523 |  | .472 |  | 0.001 |  |
| Time x Gender |  | 0.64 |  | 1,72 |  | 0.637 |  | 2.889 |  | .094 |  | 0.006 |  |
| Group x Ge |  | 0.07 |  | 1,72 |  | 0.070 |  | 0.056 |  | .814 |  | 0.006 |  |
| T x Gr x Ge |  | 0.30 |  | 1,72 |  | 0.299 |  | 1.356 |  | .248 |  | 0.003 |  |
| *Note.* Type III Sum of Squares (SS); Sample sizes were *n* = 36/37 for the open-label-placebo group and *n* = 39 for the control group; BF_01_ = evidence for the null vs. the alternative hypothesis. | | | | | | | | | | | | |  |

Table S15. Mixed ANOVAs and BF_01_’s for the two outcomes of the instrumental learning task, including gender.

| Measure & Effect | | $SS$ | | *df*‘s | | Mean Square | | $F$ | | $p$ | | $ƞ^{2}$ | |
| --- | --- | --- | --- | --- | --- | --- | --- | --- | --- | --- | --- | --- | --- |
| Reaction time |  |  |  |  |  |  |  |  |  |  |  |  |  |
| Time |  | 1.10 |  | 1,64 |  | 1.101 |  | 94.457 |  | **< .001** |  | 0.213 |  |
| Group |  | 0.01 |  | 1,64 |  | 0.009 |  | 0.167 |  | .684 |  | 0.002 |  |
| Gender |  | < 0.01 |  | 1,64 |  | 0.002 |  | 0.005 |  | .944 |  | < 0.001 |  |
| Time x Group |  | 0.01 |  | 1,64 |  | 0.007 |  | 0.066 |  | .798 |  | 0.001 |  |
| Time x Gender |  | 0.01 |  | 1,64 |  | 0.006 |  | 0.058 |  | .810 |  | 0.001 |  |
| Group x Ge |  | < 0.01 |  | 1,64 |  | 0.002 |  | 0.031 |  | .862 |  | 0.003 |  |
| T x Gr x Ge |  | 0.01 |  | 1,64 |  | 0.012 |  | 1.012 |  | .318 |  | 0.002 |  |
| Hits |  |  |  |  |  |  |  |  |  |  |  |  |  |
| Time |  | 1653.74 |  | 1,64 |  | 1653.736 |  | 2.926 |  | .092 |  | 0.009 |  |
| Group |  | 129.19 |  | 1,64 |  | 129.191 |  | 0.055 |  | .815 |  | 0.006 |  |
| Gender |  | 15.15 |  | 1,64 |  | 15.153 |  | 0.006 |  | .936 |  | < 0.001 |  |
| Time x Group |  | 644.86 |  | 1,64 |  | 644.857 |  | 1.141 |  | .289 |  | 0.003 |  |
| Time x Gender |  | 4.66 |  | 1,64 |  | 4.664 |  | 0.008 |  | .928 |  | < 0.001 |  |
| Group x Ge |  | 842.04 |  | 1,64 |  | 842.04 |  | 0.359 |  | .551 |  | 0.004 |  |
| T x Gr x Ge |  | 8.51 |  | 1,64 |  | 8.507 |  | 0.015 |  | .903 |  | < 0.001 |  |
| *Note.* Type III Sum of Squares (SS); Sample sizes were *n* = 32 for the open-label-placebo group and *n* = 36 for the control group; BF_01_ = evidence for the null vs. the alternative hypothesis. | | | | | | | | | | | | |  |

*Table S16. Mixed ANOVAs and BF_01_’s for the three outcomes of the d2 task, including gender.*

| Measure & Effect | | $SS$ | | *df*‘s | | Mean Square | | $F$ | | $p$ | | $ƞ^{2}$ | |
| --- | --- | --- | --- | --- | --- | --- | --- | --- | --- | --- | --- | --- | --- |
| CTO |  |  |  |  |  |  |  |  |  |  |  |  |  |
| Time |  | 12050.46 |  | 1,72 |  | 12050.459 |  | 57.701 |  | **< .001** |  | 0.055 |  |
| Group |  | 473.21 |  | 1,72 |  | 473.205 |  | 0.195 |  | .660 |  | 0.002 |  |
| Gender |  | 13445.62 |  | 1,72 |  | 13445.617 |  | 5.541 |  | **.021** |  | 0.062 |  |
| Time x Group |  | 11.68 |  | 1,72 |  | 11.679 |  | 0.056 |  | .814 |  | < 0.001 |  |
| Time x Gender |  | 200.35 |  | 1,72 |  | 200.350 |  | 0.959 |  | .331 |  | 0.009 |  |
| Group x Ge |  | 1824.69 |  | 1,72 |  | 1824.690 |  | 0.752 |  | .389 |  | 0.008 |  |
| T x Gr x Ge |  | 50.65 |  | 1,72 |  | 50.650 |  | 0.243 |  | .624 |  | 0.002 |  |
| E% |  |  |  |  |  |  |  |  |  |  |  |  |  |
| Time |  | 451.78 |  | 1,72 |  | 451.777 |  | 15.027 |  | **< .001** |  | 0.032 |  |
| Group |  | 4.44 |  | 1,72 |  | 4.437 |  | 0.029 |  | .865 |  | 0.003 |  |
| Gender |  | 198.59 |  | 1,72 |  | 198.592 |  | 1.302 |  | .258 |  | 0.014 |  |
| Time x Group |  | 13.05 |  | 1,72 |  | 13.045 |  | 0.434 |  | .512 |  | 0.009 |  |
| Time x Gender |  | 15.61 |  | 1,72 |  | 15.613 |  | 0.519 |  | .473 |  | 0.001 |  |
| Group x Ge |  | 98.79 |  | 1,72 |  | 98.794 |  | 0.648 |  | .424 |  | 0.007 |  |
| T x Gr x Ge |  | 0.528 |  | 1,72 |  | 0.528 |  | 0.018 |  | .895 |  | < 0.001 |  |
| CP |  |  |  |  |  |  |  |  |  |  |  |  |  |
| Time |  | 22049.803 |  | 1,72 |  | 22049.803 |  | 145.318 |  | **< .001** |  | 0.101 |  |
| Group |  | 363.975 |  | 1,72 |  | 363.975 |  | 0.150 |  | .700 |  | 0.002 |  |
| Gender |  | 4938.468 |  | 1,72 |  | 4938.468 |  | 2.029 |  | .159 |  | 0.023 |  |
| Time x Group |  | 158.499 |  | 1,72 |  | 158.499 |  | 1.045 |  | .310 |  | 7.255e-4 |  |
| Time x Gender |  | 616.607 |  | 1,72 |  | 616.607 |  | 4.064 |  | **.048** |  | 0.003 |  |
| Group x Ge |  | 4125.739 |  | 1,72 |  | 4125.739 |  | 1.695 |  | .197 |  | 0.019 |  |
| T x Gr x Ge |  | 35.578 |  | 1,72 |  | 35.578 |  | 0.234 |  | .630 |  | 1.628e-4 |  |
| *Note.* Type III Sum of Squares (SS); Sample sizes were *n* = 38 for the open-label-placebo group and *n* = 38 for the control group; CTO = number of finished target objects; E% = error percent; CP = concentration performance. | | | | | | | | | | | | |  |

*Table S17. Mixed ANOVAs and BF_01_’s for subjective effort in the five objective tasks, including gender.*

| Measure & Effect | | $SS$ | | *df*‘s | | Mean Square | | $F$ | | $p$ | | $ƞ^{2}$ | |  |
| --- | --- | --- | --- | --- | --- | --- | --- | --- | --- | --- | --- | --- | --- | --- |
| Stroop task |  |  |  |  |  |  |  |  |  |  |  |  |  |  |
| Time |  | 510.31 |  | 1,73 |  | 510.305 |  | 1.520 |  | .222 |  | 0.006 |  |  |
| Group |  | 94.54 |  | 1,73 |  | 94.540 |  | 0.131 |  | .718 |  | 0.001 |  |  |
| Gender |  | 958.35 |  | 1,73 |  | 958.348 |  | 1.327 |  | .253 |  | 0.012 |  |  |
| Time x Group |  | 225.97 |  | 1,73 |  | 225.968 |  | 0.673 |  | .415 |  | 0.003 |  |  |
| Time x Gender |  | 446.35 |  | 1,73 |  | 446.350 |  | 1.330 |  | .253 |  | 0.006 |  |  |
| Group x Ge |  | 4.28 |  | 1,73 |  | 4.278 |  | 0.006 |  | .939 |  | < 0.001 |  |  |
| T x Gr x Ge |  | 85.11 |  | 1,73 |  | 85.106 |  | 0.254 |  | .616 |  | 0.001 |  |  |
| 2-back task |  |  |  |  |  |  |  |  |  |  |  |  |  |  |
| Time |  | 4224.24 |  | 1,71 |  | 4224.236 |  | 26.532 |  | **< .001** |  | 0.054 |  |  |
| Group |  | 224.36 |  | 1,71 |  | 224.357 |  | 0.273 |  | .603 |  | 0.003 |  |  |
| Gender |  | 1758.01 |  | 1,71 |  | 1758.005 |  | 2.136 |  | .148 |  | 0.022 |  |  |
| Time x Group |  | 215.38 |  | 1,71 |  | 215.381 |  | 1.353 |  | .249 |  | 0.003 |  |  |
| Time x Gender |  | 1197.55 |  | 1,71 |  | 1197.547 |  | 7.522 |  | **.008** |  | 0.015 |  |  |
| Group x Ge |  | 1511.92 |  | 1,71 |  | 1511.916 |  | 1.837 |  | .180 |  | 0.019 |  |  |
| T x Gr x Ge |  | 43.81 |  | 1,71 |  | 43.808 |  | 0.275 |  | .602 |  | 0.005 |  |  |
| Dual task |  |  |  |  |  |  |  |  |  |  |  |  |  |  |
| Time |  | 1039.01 |  | 1,73 |  | 1039.008 |  | 3.226 |  | .077 |  | 0.013 |  |  |
| Group |  | 811.51 |  | 1,73 |  | 811.510 |  | 1.167 |  | .284 |  | 0.010 |  |  |
| Gender |  | 1317.86 |  | 1,73 |  | 1317.858 |  | 1.895 |  | .173 |  | 0.017 |  |  |
| Time x Group |  | 37.63 |  | 1,73 |  | 37.627 |  | 0.117 |  | .733 |  | 0.004 |  |  |
| Time x Gender |  | 246.46 |  | 1,73 |  | 246.460 |  | 0.765 |  | .385 |  | 0.003 |  |  |
| Group x Ge |  | 3.05 |  | 1,73 |  | 3.048 |  | 0.004 |  | .947 |  | < 0.001 |  |  |
| T x Gr x Ge |  | 63.54 |  | 1,73 |  | 63.541 |  | 0.197 |  | .658 |  | 0.008 |  |  |
| Learning task |  |  |  |  |  |  |  |  |  |  |  |  |  |  |
| Time |  | 2308.89 |  | 1,69 |  | 2308.893 |  | 4.818 |  | .032 |  | 0.028 |  |  |
| Group |  | 570.95 |  | 1,69 |  | 570.945 |  | 0.843 |  | .362 |  | 0.007 |  |  |
| Gender |  | 144.61 |  | 1,69 |  | 144.610 |  | 0.214 |  | .645 |  | 0.002 |  |  |
| Time x Group |  | 2.82 |  | 1,69 |  | 2.817 |  | 0.006 |  | .939 |  | < 0.001 |  |  |
| Time x Gender |  | 76.76 |  | 1,69 |  | 76.758 |  | 0.160 |  | .690 |  | 0.009 |  |  |
| Group x Ge |  | 2.06 |  | 1,69 |  | 2.055 |  | 0.003 |  | .956 |  | 0.002 |  |  |
| T x Gr x Ge |  | 122.24 |  | 1,69 |  | 122.236 |  | 0.255 |  | .615 |  | 0.001 |  |  |
| d2 task |  |  |  |  |  |  |  |  |  |  |  |  |  |  |
| Time |  | 6617.46 |  | 1,71 |  | 6617.459 |  | 24.512 |  | **< .001** |  | 0.075 |  |  |
| Group |  | 69.04 |  | 1,71 |  | 69.042 |  | 0.079 |  | .779 |  | 0.007 |  |  |
| Gender |  | 542.41 |  | 1,71 |  | 542.406 |  | 0.622 |  | .433 |  | 0.006 |  |  |
| Time x Group |  | 42.19 |  | 1,71 |  | 42.191 |  | 0.156 |  | .694 |  | 0.004 |  |  |
| Time x Gender |  | 217.30 |  | 1,71 |  | 217.301 |  | 0.805 |  | .373 |  | 0.002 |  |  |
| Group x Ge |  | 6.48 |  | 1,71 |  | 6.483 |  | 0.007 |  | .932 |  | < 0.001 |  |  |
| T x Gr x Ge |  | 193.41 |  | 1,71 |  | 193.407 |  | 0.716 |  | .400 |  | 0.002 |  |  |
| *Note.* Type III Sum of Squares (SS); BF_01_ = evidence for the null vs. the alternative hypothesis. | | | | | | | | | | | | | | |

Table S18. Mixed ANOVAs and BF_01_’s for subjective satisfaction in the five objective tasks, including gender.

| Measure & Effect | | $SS$ | | *df*‘s | | Mean Square | | $F$ | | $p$ | | $ƞ^{2}$ | |
| --- | --- | --- | --- | --- | --- | --- | --- | --- | --- | --- | --- | --- | --- |
| Stroop task |  |  |  |  |  |  |  |  |  |  |  |  |  |
| Time |  | 0.15 |  | 1,73 |  | 0.147 |  | 0.003 |  | .986 |  | < 0.001 |  |
| Group |  | 480.08 |  | 1,73 |  | 480.082 |  | 0.915 |  | .342 |  | 0.006 |  |
| Gender |  | 12.53 |  | 1,73 |  | 12.525 |  | 0.024 |  | .878 |  | 0.001 |  |
| Time x Group |  | 2.67 |  | 1,73 |  | 2.671 |  | 0.006 |  | .941 |  | < 0.001 |  |
| Time x Gender |  | 597.13 |  | 1,73 |  | 597.128 |  | 1.238 |  | .270 |  | 0.008 |  |
| Group x Ge |  | 267.77 |  | 1,73 |  | 267.772 |  | 0.510 |  | .477 |  | 0.004 |  |
| T x Gr x Ge |  | 254.56 |  | 1,73 |  | 254.564 |  | 0.528 |  | .470 |  | 0.003 |  |
| 2-back task |  |  |  |  |  |  |  |  |  |  |  |  |  |
| Time |  | 3622.37 |  | 1,71 |  | 3622.374 |  | 9.460 |  | 0.003 |  | 0.049 |  |
| Group |  | 393.88 |  | 1,71 |  | 393.878 |  | 0.670 |  | 0.416 |  | 0.005 |  |
| Gender |  | 289.02 |  | 1,71 |  | 289.018 |  | 0.492 |  | .485 |  | 0.004 |  |
| Time x Group |  | 644.34 |  | 1,71 |  | 644.342 |  | 1.683 |  | .199 |  | 0.009 |  |
| Time x Gender |  | 143.29 |  | 1,71 |  | 143.294 |  | 0.374 |  | .543 |  | 0.002 |  |
| Group x Ge |  | 63.06 |  | 1,71 |  | 63.061 |  | 0.107 |  | .744 |  | 0.008 |  |
| T x Gr x Ge |  | 476.60 |  | 1,71 |  | 476.600 |  | 1.245 |  | .268 |  | 0.006 |  |
| Dual task |  |  |  |  |  |  |  |  |  |  |  |  |  |
| Time |  | 568.26 |  | 1,73 |  | 568.259 |  | 2.429 |  | .123 |  | 0.008 |  |
| Group |  | 540.54 |  | 1,73 |  | 540.537 |  | 0.708 |  | .403 |  | 0.007 |  |
| Gender |  | 47.87 |  | 1,73 |  | 47.873 |  | 0.063 |  | .803 |  | 0.006 |  |
| Time x Group |  | 907.95 |  | 1,73 |  | 907.950 |  | 3.882 |  | .053 |  | 0.012 |  |
| Time x Gender |  | 238.23 |  | 1,73 |  | 238.233 |  | 1.018 |  | .316 |  | 0.003 |  |
| Group x Ge |  | 279.76 |  | 1,73 |  | 279.764 |  | 0.366 |  | .547 |  | 0.004 |  |
| T x Gr x Ge |  | 9.10 |  | 1,73 |  | 9.101 |  | 0.039 |  | .844 |  | 0.001 |  |
| Learning task |  |  |  |  |  |  |  |  |  |  |  |  |  |
| Time |  | 88.28 |  | 1,69 |  | 88.284 |  | 0.300 |  | .586 |  | 0.001 |  |
| Group |  | 1192.91 |  | 1,69 |  | 1192.909 |  | 1.768 |  | .188 |  | 0.017 |  |
| Gender |  | 137.38 |  | 1,69 |  | 137.380 |  | 0.204 |  | .653 |  | 0.002 |  |
| Time x Group |  | 1380.99 |  | 1,69 |  | 1380.986 |  | 4.693 |  | **.034** |  | 0.019 |  |
| Time x Gender |  | 409.87 |  | 1,69 |  | 409.866 |  | 1.393 |  | .242 |  | 0.006 |  |
| Group x Ge |  | 814.08 |  | 1,69 |  | 814.076 |  | 1.207 |  | .276 |  | 0.011 |  |
| T x Gr x Ge |  | 56.71 |  | 1,69 |  | 56.707 |  | 0.193 |  | .662 |  | 0.008 |  |
| d2 |  |  |  |  |  |  |  |  |  |  |  |  |  |
| Time |  | 1416.59 |  | 1,71 |  | 1416.591 |  | 4.836 |  | **.031** |  | 0.025 |  |
| Group |  | 2123.05 |  | 1,71 |  | 2123.053 |  | 4.952 |  | **.029** |  | 0.037 |  |
| Gender |  | 88.84 |  | 1,71 |  | 88.842 |  | 0.207 |  | .650 |  | 0.002 |  |
| Time x Group |  | 1054.43 |  | 1,71 |  | 1054.428 |  | 3.599 |  | .062 |  | 0.018 |  |
| Time x Gender |  | 302.95 |  | 1,71 |  | 302.949 |  | 1.034 |  | .313 |  | 0.005 |  |
| Group x Ge |  | 5.23 |  | 1,71 |  | 5.230 |  | 0.012 |  | .912 |  | < 0.001 |  |
| T x Gr x Ge |  | 1420.92 |  | 1,71 |  | 1420.915 |  | 4.851 |  | **.031** |  | 0.025 |  |
| *Note.* Type III Sum of Squares (SS); BF_01_ = evidence for the null vs. the alternative hypothesis. | | | | | | | | | | | | | |

Table S19. Welch’s t-tests and BF_01_’s for subjective change in the five objective tasks, including gender.

| Measure | | $SS$ | $F$ | *df‘s* | | $p$ | | $ƞ^{2}$ |
| --- | --- | --- | --- | --- | --- | --- | --- | --- |
| Stroop task |  |  |  |  |  |  |  |  |
| Group |  | 0.53 | 0.002 | 1,73 |  | .964 |  | < 0.001 |
| Gender |  | 143.73 | 0.542 | 1,73 |  | .464 |  | 0.007 |
| Group x Ge |  | 102.90 | 0.388 | 1,73 |  | .535 |  | 0.005 |
| 2-back task |  |  |  |  |  |  |  |  |
| Group |  | 435.77 | 1.763 | 1,72 |  | .188 |  | 0.023 |
| Gender |  | 392.76 | 1.589 | 1,72 |  | .212 |  | 0.021 |
| Group x Ge |  | 219.05 | 0.886 | 1,72 |  | .350 |  | 0.012 |
| Dual task |  |  |  |  |  |  |  |  |
| Group |  | 176.65 | 0.802 | 1,73 |  | .374 |  | 0.011 |
| Gender |  | 147.29 | 0.668 | 1,73 |  | .416 |  | 0.009 |
| Group x Ge |  | 119.54 | 0.542 | 1,73 |  | .464 |  | 0.007 |
| Learning task |  |  |  |  |  |  |  |  |
| Group |  | 13.31 | 0.064 | 1,71 |  | .801 |  | 0.008 |
| Gender |  | 236.82 | 1.140 | 1,71 |  | .289 |  | 0.016 |
| Group x Ge |  | 72.36 | 0.348 | 1,71 |  | .557 |  | 0.005 |
| d2 task |  |  |  |  |  |  |  |  |
| Group |  | 0.27 | 0.001 | 1,71 |  | .970 |  | < 0.001 |
| Gender |  | 695.42 | 3.812 | 1,71 |  | .055 |  | 0.051 |
| Group x Ge |  | 33.27 | 0.182 | 1,71 |  | .671 |  | 0.002 |
| *Note.* Type III Sum of Squares (SS); BF_01_ = evidence for the null vs. the alternative hypothesis. | | | | | | | | |
